# Nonlinear Stress‐Induced Transformations in Collagen Fibrillar Organization, Disorder and Strain Mechanisms in the Bone‐Cartilage Unit

**DOI:** 10.1002/advs.202407649

**Published:** 2024-11-11

**Authors:** Waqas Badar, Sheetal R. Inamdar, Peter Fratzl, Tim Snow, Nicholas J. Terrill, Martin M. Knight, Himadri S. Gupta

**Affiliations:** ^1^ Centre for Bioengineering and School of Engineering and Materials Science Queen Mary University of London London E1 4NS UK; ^2^ Max Planck Institute of Colloids and Interfaces Research Campus Golm 14424 Potsdam Germany; ^3^ Diamond Light Source Harwell Science Campus Harwell OX11 0DE UK

**Keywords:** 3D diffraction modelling, bone‐cartilage interface, collagen fibrils, nanoscale mechanics, small‐angle x‐ray scattering

## Abstract

By developing a 3D X‐ray modeling and spatially correlative imaging method for fibrous collagenous tissues, this study provides a comprehensive mapping of nanoscale deformation in the collagen fibril network across the intact bone‐cartilage unit (BCU), whose healthy functioning is critical for joint function and preventing degeneration. Extracting the 3D fibril structure from 2D small‐angle X‐ray scattering before and during physiological compression reveals of dominant deformation modes, including crystallinity transitions, lateral fibril compression, and reorientation, which vary in a coupled, nonlinear, and correlated manner across the cartilage‐bone interface. A distinct intermolecular arrangement of collagen molecules, and enhanced molecular‐level disorder, is found in the cartilage (sliding) surface region. Just below, fibrils accommodate tissue strain by reorientation, which transitions molecular‐level kinking or loss of crystallinity in the deep zone. Crystalline fibrils laterally shrink far more (20×) than they contract, possibly by water loss from between tropocollagen molecules. With the calcified plate acting as an anchor for surrounding tissue, a qualitative switch occurs in fibrillar deformation between the articular cartilage and calcified regions. These findings significantly advance this understanding of the complex, nonlinear ultrastructural dynamics at this critical interface, and opens avenues for developing targeted diagnostic and therapeutic strategies for musculoskeletal disorders.

## Graphical abstract

The ultrastructural mechanisms enabling physiological compression in cartilage/bone interfaces in joints are explored using X‐ray nanomechanical imaging and 3D modeling of the hydrated collagen fibril/proteoglycan nanostructure. The approach reveals synergistic and microscale spatially‐graded stress‐induced interactions between hidden modes like fibrillar compaction, fluid flow, crystallinity transitions, and reorientation. These molecular‐to‐microscale biophysical mechanisms advance fundamental understandings of joint function and pathological degeneration.

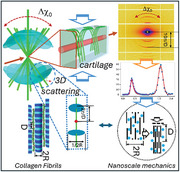

## 1 Introduction

**Figure 1:**
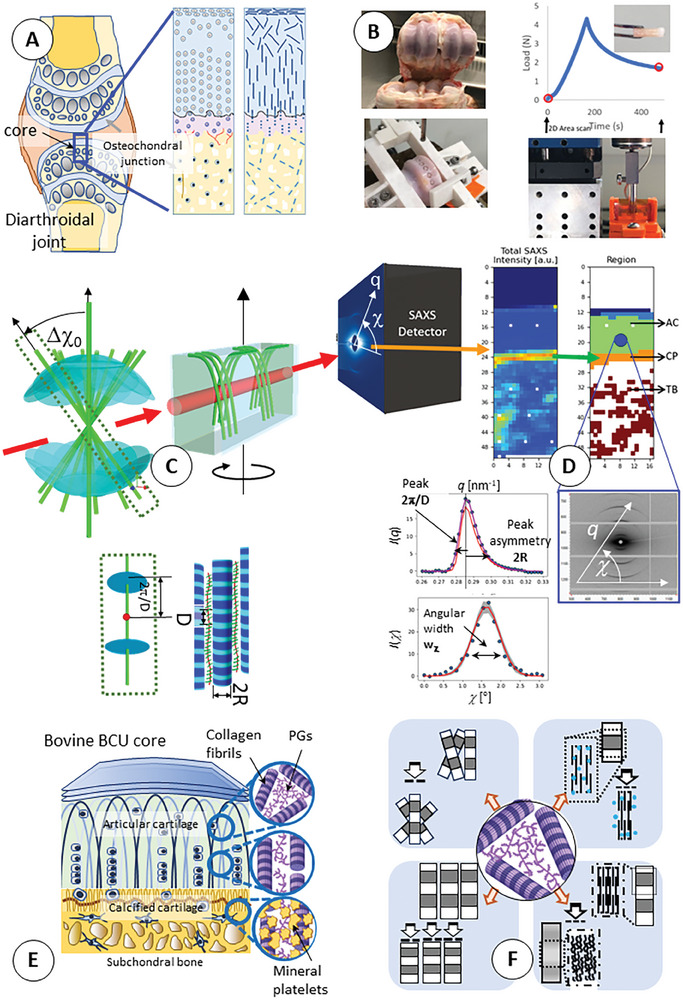
Hierarchical structure of cartilage and experimental setup: A) left: Schematic of diarthrodial joint; blue rectangle region of BCU core; right: Graded BCU structure at cellular (left) and ECM (right) level B) left: Bovine metacarpophalangeal joint; coring setup below; right: schematic stress‐relaxation curve of BCU, mounted in in situ compression tester (below). C) 3D fiber‐diffraction model for collagen fibrils in the graded BCU. Top: 3D scattering intensity as sum of individual fibril scattering at different orientations; red arrow: X‐ray going through arcade‐like Benninghof structure (green lines: collagen fibrils). Fiber symmetry assumption around vertical axis. Bottom: Individual fibril with 3D meridional pancake‐shaped intensity distributions (blue), spaced by 2π/D; radius inversely proportional to fibril diameter 2R. D) Top: SAXS intensity maps and anatomical classification. Below: 2D SAXS deep zone pattern and example fits of 3D X‐ray model to radial and azimuthal profiles. E) Left: ECM schematic of BCU; insets showing fibril structure variation. Right: Potential unknown fibrillar deformation mechanisms including (clockwise top left): angular reorientation, fibrillar lateral compaction with intramolecular water expulsion, intrafibrillar molecular rearrangements and axial compression.

The bone‐cartilage unit (BCU) is a critical component of diarthrodial joints, enabling pain‐free movement and frictionless articulation.^[^ ^1^ ^]^ Structural and mechanical deterioration in the BCU, often initiated through mechanical trauma, are believed to be critical factors in the progression of widespread musculoskeletal disorders like osteoarthritis (OA),^[^ ^2^ ^]^ which affects a significant and growing fraction of the global populace (>500 million people worldwide and increasing^[^ ^3^ ^]^). The BCU exhibits gradients in matrix composition, structure, and mechanics across its extent, with a relatively soft (0.5–10 MPa) fibrous hydrogel (articular cartilage) interlocking with an underlying hard calcified tissue (calcified cartilage and subchondral bone). Spreading the mechanical load and reducing strain, linked to biochemical signaling across the BCU, is essential for everyday functionality. However, the hierarchical structural organization of the BCU tissue matrix (**Figure** **1**A) means the in vivo biomechanics at the ultrastructural level are challenging to measure. An essential component of the ultrastructural organization is the Type II‐ and I‐ collagen fibril network, which interpenetrates with a hydrated, gel‐like extrafibrillar matrix (mainly negatively charged glycosaminoglycans) in the articular cartilage,^[^ ^4^ ^]^ transitions to stiffer mineralized fibrils in the calcified cartilage, and changes to mineralized Type I collagen in the underlying subchondral bone. How this collagen network responds to physiological loading is unclear, including the mechanisms by which the graded fibrillar structure accommodates a spatially varying strain, the deformation modes which arise at the extra‐ and intrafibrillar level, and the way the compositional and biochemical transition to the calcified interface modulates these mechanisms.

In more detail, the fibers (micron scale) in articular cartilage form an arcade‐like network (the Benninghof structure;^[^ ^5^ ^]^ Figure 1A,E). Fibers run parallel to the joint surface, and smoothly change orientation to perpendicular to the calcified interface. Within fibers, the extracellular matrix (ECM) exists in mechanical equilibrium between a swelling pressure exerted by proteoglycans and tensile resistance to this swelling provided by the Type‐II collagen fibrillar meshwork (Figure 1E). Collagen fibrils are thus in a state of pre‐strain in vivo, which has been shown to change under compression or enzymatic digestion.^[^ ^6^ ^]^ Finite‐element poroelastic models of cartilage structure,^[^ ^7^ ^]^ considering the ECM as a multi‐phase material and incorporating fibril reinforcement and orientation, have successfully modelled tissue‐level mechanics, and deterioration due to osteoarthritis. However, comparisons to fibril‐level experimental mechanics are limited. At the 1–10 µm fiber level, absorption contrast X‐ray tomographic mapping across the bone‐cartilage interface has demonstrated the existence of high shear strains in the bone cells in the calcified part of the interface.^[^ ^8^ ^]^ Second harmonic generation microscopy has probed reorientation of collagen under strain.^[^ ^9^ ^]^ Ultrastructural changes in collagen orientation^[^ ^10^, ^11^ ^]^ and matrix composition^[^ ^12^ ^]^ in osteoarthritic patients have been identified using X‐ray scattering. Recently, phase‐contrast tomography with in situ loading has demonstrated cellular compaction and fiber angle reorientation in the articular cartilage.^[^ ^13^ ^]^ However, such fibers (1–10 µm) are comprised of nanoscale (10–100 nm diameter) collagen fibrils,^[^ ^14^ ^]^ and very limited information is available on the matrix biomechanics of this underlying fibrillar‐network in the intact bone‐cartilage unit. There is thus a clear need for full‐field quantitative nanoscale imaging of the fibrillar‐level mechanics across the cartilage and underlying bone under physiological compression in situ, especially in identifying new modes of deformation arising out of the coupled, hierarchical fibrous hydrogel structure.

The fibril structure is hierarchical (tropocollagen molecules form an axially staggered arrangement at the microfibrillar level), and exists on a spatial continuum, where potentially, intra‐ and interfibrillar disorder could act as modulating factors, linked to stress, enzymatic perturbation (like protease‐assisted fibrillar degradation), and change interactions of the fibrils with the other macromolecules. Using structural probes like small‐angle X‐ray scattering (SAXS) and wide‐angle X‐ray diffraction (WAXD) prior work has identified anatomically variable fibril orientation^[^ ^15^ ^]^ depending on in vivo loads^[^ ^16^ ^]^ and structural changes in osteoarthritic lesions,^[^ ^10^ ^]^ and increases in axial fibril pre‐strain from the superficial to deep zones which is disrupted in loading and protease‐mediated synthetic ageing.^[^ ^6^ ^]^ Recently, we showed that undeformed BCU cores from bovine joints exhibited differences in pre‐strain between calcified plate and the underlying trabecular bone.^[^ ^17^ ^]^ These studies are, however, limited in the following aspects – 1) most do not consider the cartilage and bone mechanics together, 2) full‐field nanomechanical imaging or mapping (needed for a spatially‐variable matrix structure like the BCU) is missing, 3) the analysis of the fibrillar response do not consider the 3D fibrillar structure crucial for in vivo function, and 4) the collagen network is considered as essentially thin 1D collagen fibrils at different levels of pre‐strain, not considering other modes of deformation that may be relevant for such soft biopolymer networks. Point‐ and pencil‐beam imaging techniques like microfocus SAXS mapping combined with large‐scale area scans open new vistas in the analysis of hierarchical biological structural tissues but need equally development of quantitative analysis methods to identify intrinsic 3D supramolecular structure and anatomical and tissue‐level spatial variations in such structures in health and disease.

Directly tackling these considerations, here we report the first full‐field mapping of the nanoscale deformation in the fibrillar network across the entire intact bone‐cartilage unit. Our work combines a new 3D modelling technique to extract the fibril collagen structure from 2D SAXS patterns, with spatial correlative imaging to explore how different modes of nanoscale deformation interact with each other across the articular cartilage and identify transitions across the calcified interface. Notably, our approach enables the identification of dominant deformation modes in the ordered fibrillar network (intra‐ and interfibrillar strain heterogeneity, crystallinity transitions, lateral strain at the fibril level, reorientation). These phenomena go beyond prior measures of fibrillar mechanics like shifts in axial periodicity (fibril strain) used to describe tissue nanomechanics. We find that these changes vary in a coupled, nonlinear, and correlated manner across the cartilage‐bone interface. By quantifying the complex, nonlinear dynamics of the collagen fibril network in situ in the BCU, our work provides fundamental new insights into the nanoscale biophysics of this prototypical biomedically crucial interface. A characteristic of our spatially resolved technique and modelling is that our results do not solely speak to the ultrastructural level but also show the interaction between supramolecular deformation modes and the larger scale microscale mechanisms in an integrated manner.

## 2 Results and Discussion

### 2.1 A 3D Structural Model of the Fibril Enables Nano‐ and Microscale Mechanical Analysis

**Figure 2:**
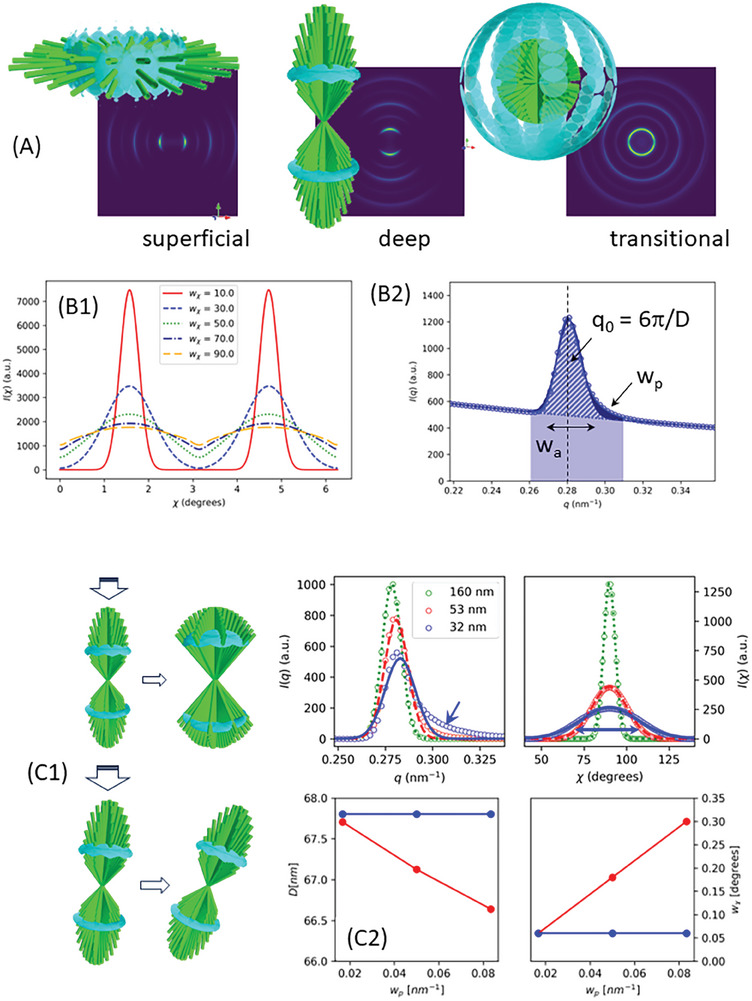
3D diffraction model for fibril scattering in articular cartilage. A) Top: 3D schematic renderings of fibrils (green) and reciprocal space meridional ellipses blue) for (left) superficial (middle) deep and (right) transitional zones. Below: predicted 2D SAXS patterns. The angular spaces between fibers are for visual clarity in showing meridional ellipses. B1) Simulated I(χ) patterns for deep zone fibrils with increasing w_χ_. B2) Simulated I(q) curve indicating experimental parameters like D, w_a_, w_p_, collagen peak intensity (dashed) and total SAXS intensity (blue‐shaded region). C1) Top: Load‐induced splaying of a conical distribution versus (bottom) tilting for misaligned distributions in response to vertical compressive load. C2) Error in D‐period (left) and angular misalignment w_χ_ estimation with decrease in fibril radius and standard Gaussian profiles used for I(q) and I(χ); blue line: true values; red line: fitted (incorrect) values.

Cores of bone‐cartilage tissue were extracted from bovine metacarpophalangeal joints, mounted in a microcompression tester, and scanned with synchrotron microfocus SAXS (Beamline I22, Diamond Light Source, Harwell) in uncompressed and compressed states with three different initial loading rates (see Experimental Section). From the 2D SAXS raster‐scan, we implemented a zonal tissue‐classification system for articular cartilage, calcified plate, and trabecular bone (right, Figure 1D), based on a metric combining angular peak position, width, and SAXS intensity (Experimental Section). This initial step is essential to identify the fibril orientation distribution type in each zone. Figure 1D shows the meridional scattering peaks from a single collagen fibril, whose location in reciprocal space enables extraction of the fibrillar axial stagger (D‐period). The axial fibrillar strain is directly proportional to changes in D‐period and has two components. The first component is a thermodynamic eigenstrain, mostly due to hydration (potentially modulated by local osmotic pressure^[^ ^18^ ^]^), which may vary due to changes in physical chemistry (specifically the competition for water between collagen and proteoglycans) across the tissue. The second component is the elastic strain due to load.^[^ ^6^ ^]^ Figures 1C and 2A show how, if fibrils are oriented around a central axis (normal to the joint surface), as occurs in the deep zone, a 3D conical distribution is appropriate (also in the calcified plate). However, the fibrils in the superficial zone are perpendicular to this axis, and as a result, a ring‐like distribution is required (Figure 2A). The residual layer with no preferred orientation (classified as transitional by us, but much thinner (≈10–20% cartilage thickness) than the literature‐reported transitional zone; ≈40–60% thickness^[^ ^4^ ^]^) is assigned a 3D isotropic distribution (Figure 2A). It is thinner because our definition of the deep zone allows wide angular distributions and accommodates points which otherwise might have been labelled as transitional. These distributions account for the averaging of fibril orientations as probed by a single beam path (Figure 1C; center) across the full thickness of the sample. While their parameters (principally angular width) change within each zone, the qualitative function type is constant, and mathematical details are given in Supporting Information (Model).

In the second step, the SAXS pattern for each beam path is calculated by convolving the 3D reciprocal space scattering from a single fibril at an arbitrary orientation with the normalized angular distribution weight and extracting the intersection of the resultant 3D scattering with the Ewald sphere (Experimental Section and Supporting Information: Model). **Figure** **2** summarizes aspects of the model with simulated SAXS patterns in Figure 2A. These show the expected change in orientation and alignment across zones. The strong 3^rd^ meridional peak was used for fitting to the model. The broadening of I(χ) by increasing the width parameter w_χ_ of the spherical orientation distribution is shown in Figure 2B1. The SAXS intensity and collagen peak intensity are extracted as shown in Figure 2B2. Due to the coring geometry used (normal to the cartilage surface: Figure 1B) along with the loading along this axis, we make a fiber‐symmetry assumption of fibril orientation around this axis (Figure 1C, schematic), leading to a SAXS signal from an aggregate of fibrils in a conical distribution oriented vertically (Figure 2C1, left). Under load, such a distribution is expected to remain vertical but splays out (increasing w_χ_). For initially misaligned distributions, a tilting further out of plane is expected (Figure 2C1, right). This assumption was found experimentally to be well‐fulfilled from for the low‐ and medium strain‐rate groups but less for the high strain rate group, which also experienced a higher strain due to an experimental issue; this may be a factor when considering deviations of the high‐strain rate group behavior from the other groups. Simulations of the model will also test these assumptions later (Figure S9, Supporting Information).

When considering the experimental radial I(*q*) and azimuthal I(*χ*) plots, the need for the 3D model is made evident in Figure 2C2: due to the finite fibril diameter, a broadening of I(χ) from a narrowing of fibril diameter could incorrectly assigned to an increasing angular misalignment w_χ_. Similarly, identifying the correct value of the collagen D‐period requires correcting for the asymmetric right‐skew in the radial profile arising from small fibril diameter. Such a skew would bias Gaussian estimates (as done previously^[^ ^17^, ^19^ ^]^) of the D‐period toward lower values (Figure 2C2, lower plot, red symbols, and line), and would increase as the fibril radius decreases; however the effect is not so strong for Type I collagen fibrils (diameters 100–200 nm) compared to Type II fibrils (40–80 nm) as seen in Figure 2C2 (top left). The percentage error arising out of this bias is ≈1–2% for D‐period (Figure S12, Supporting Information). This error is of the order, or larger than, the experimental changes in D‐period during compression, and hence the use of the diffraction model is essential rather than previously used empirical Gaussian functions.

Using this model, we extracted the key collagen nanostructural parameters for each beam path: average *D*‐period (linked to fibrillar strain as discussed above), average scattering power I_0_, variability in ΔD, fibril diameter, and the direction and degree of alignment w_χ_, both before and after loading (Experimental Section*: 3D diffraction model* and Section S1, Supporting Information). Estimates of tissue strain were calculated directly from the images and used to estimate percentage changes and multi‐modal strains due to loading.

### 2.2 2D Full‐Field Nanoscale Imaging Before and After Loading

**Figure 3:**
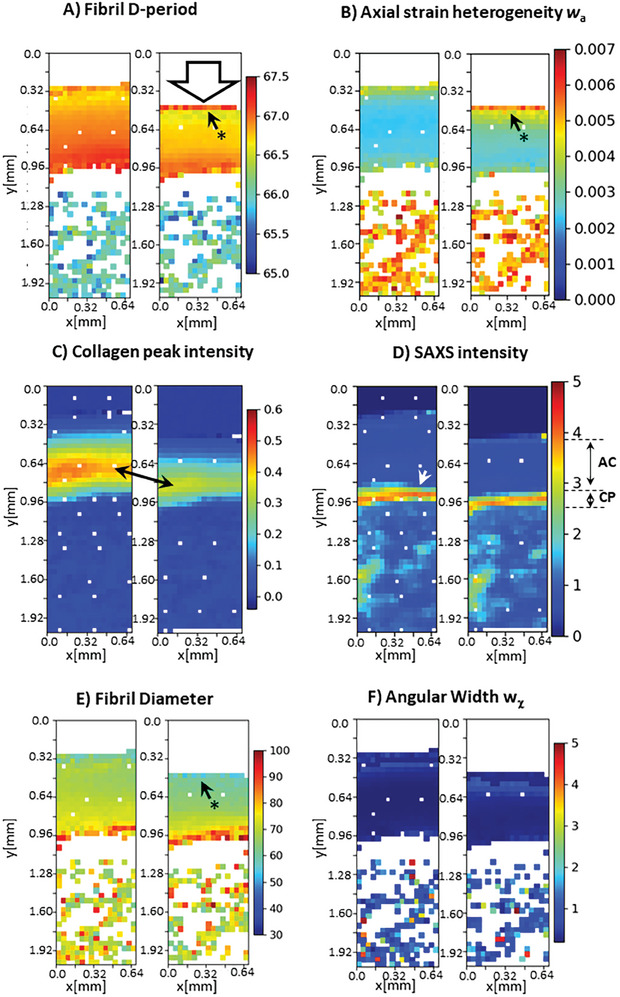
Color‐maps of SAXS ultrastructural parameters across the BCU. Parameters: A) collagen D‐period B) axial strain heterogeneity C) SAXS intensity from meridional scattering alone, D) total SAXS intensity, E) fibril diameter and F) angular width w_χ_. The sample is oriented with the superficial (sliding) surface at the top and the underlying trabecular bone at the bottom. For each image pair from A–F), left: before compression, right: compressed state; arrow in A) denotes direction of compression. Each pixel is 40 × 40 µm^2^ in dimension. Anatomical regions can be distinguished in C,D), with (uncompressed) articular cartilage between 0.32–0.80 mm, calcified plate between 0.80 and 0.96 mm, and trabecular bone below 0.96 mm. AC/CP bidirectional arrows in D) represent the length of the AC and CP zones, calculated on a laterally resolved basis for greater precision.

Under compression, 2D full‐field imaging of the collagenous matrix (**Figure** **3**) exhibits coupled complementary deformation modes – loss of fibrillar pre‐tension, lateral compaction, strain heterogeneity and fibril rotation – which vary continuously from the superficial zone through to the underlying bone. The principal scattering intensity markers, which resolve intra‐cartilage structure (collagen intensity; Figure 3c) and calcified tissue (total intensity; Figure 3d), show compression‐induced reductions in only the collagen intensity in the cartilage (bidirectional arrow, Figure 3c). Qualitatively, in the soft cartilage tissue, clear reductions in the elevated cartilage D‐period are visible in the 2D color‐map, along with colocalised increases in strain heterogeneity, reduced fibrillar diameter and alignment (* in Figure 3a,c,e). A local increase in D‐period and w_a_, along with a reduction in fibril diameter, at the top of the superficial zone is also visible (* with arrow in Figure 3a,b,e). The underlying trabecular bone shows minimal changes, being protected by the calcified plate comprising both cartilage and compact bone. As expected, most of the strain is in the cartilage, and clear demarcation of the AC/CP boundary (Figure 3d), which shifts only minimally, enables the articular cartilage strain to be calculated on a per‐sample basis, reducing error from inter‐sample variability.

### 2.3 Non‐Gaussian Shape Changes Indicating Nonlinear Changes within Tissue Regions

By grouping this multifaceted fibrillar mechanical response into anatomically resolved statistical distributions with averages and standard deviations, initial underlying trends (Figures S1–S3, Supporting Information) show that fibrillar D‐period decreases *both* in cartilage and the calcified plate to comparable levels as marked by the peak position. Such a similar shift is not expected, considering that the plate has much higher mineralization and stiffness, reducing potential deformability. Further, the transition from a bimodal (unstressed) to a unimodal distribution indicates more complex underlying biophysics to be explored below.

Interestingly, compression reduces fibrillar radius in articular cartilage but *increases* slightly in the calcified plate, and (in opposition to the D‐period) the compressed state shows a bimodal distribution. In cartilage, the fibrillar strain heterogeneity and angular misalignment increase on compression, but minimal changes are observed in the calcified compartments. The distributions are, however, averaged across tissue regions and do not exploit the microscopic resolution of X‐ray mapping, requiring unbiased spatially resolved protocols to be developed.

### 2.4 Fibrillar Mechanics Shows Multimodal Anisotropic Response Over Two Orders of Magnitude

**Figure 4:**
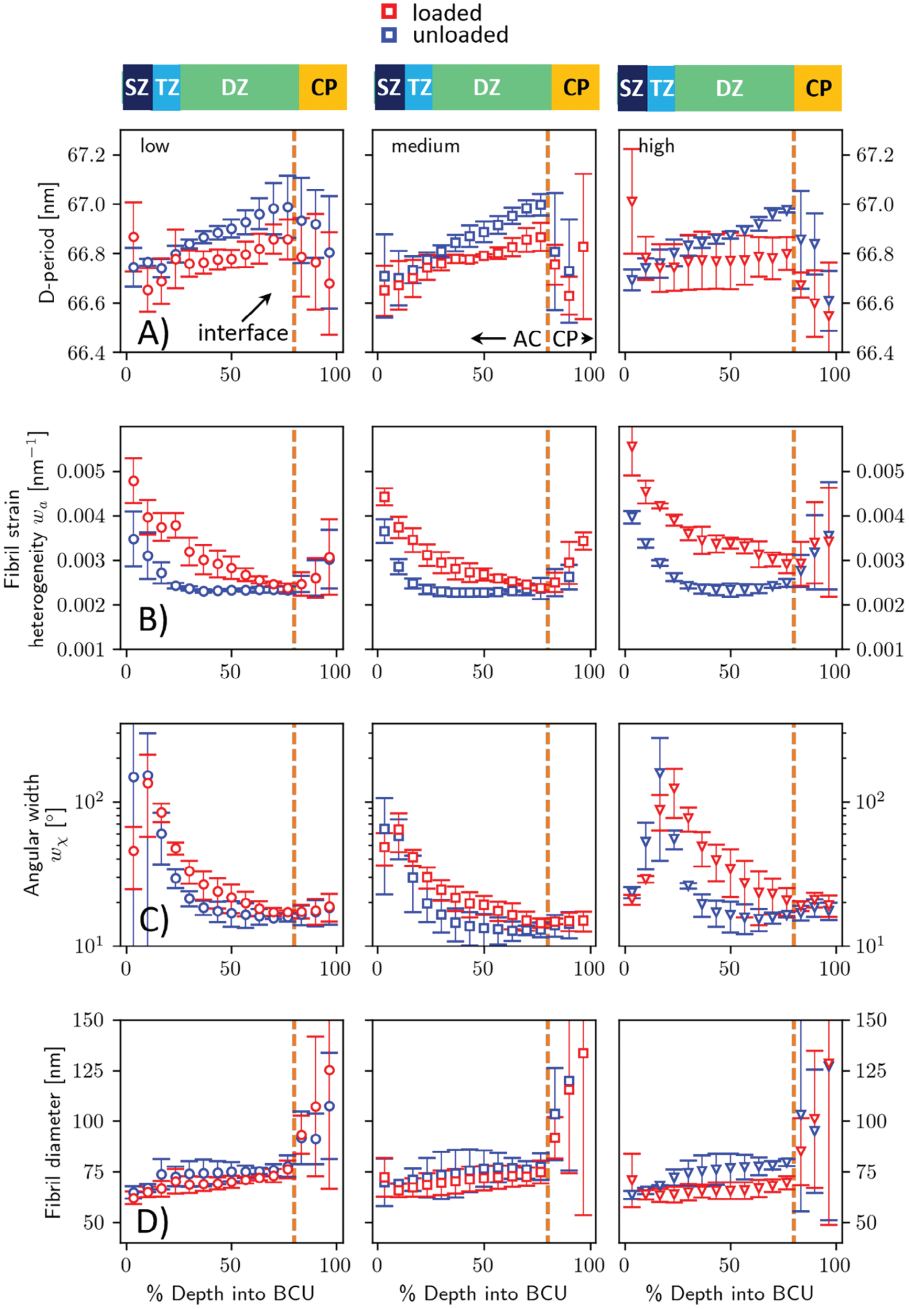
Spatial gradients in A) D‐period, B) strain heterogeneity, C) angular dispersion, and D) fibril diameter, as a function of distance from the AC/CP interface (dashed line). Columns correspond to the three strain‐rates/strains groups. Color code: blue: unloaded; red: compressed. Symbols classified by strain‐rate of load step – circles: low; squares: medium; triangles: high. Symbols with error bars: binned data averaged over strain‐rate group (*n* = 3 samples per group).

**Figure 5:**
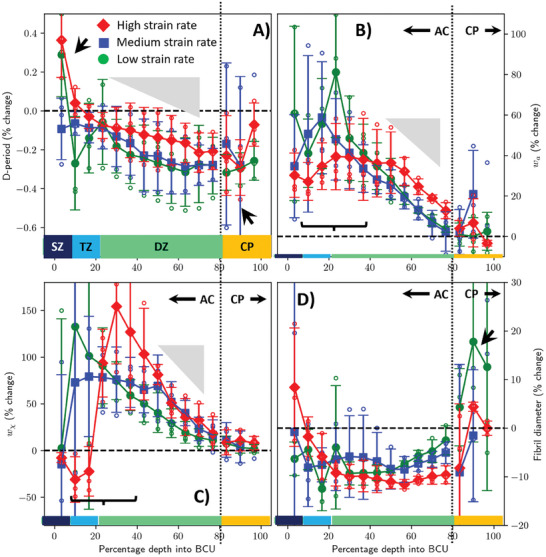
Spatial variation of magnitudes of relative fibrillar deformation modes across the bone‐cartilage unit: Percentage change in A) D‐period B) fibril strain heterogeneity *w*_a_ C) angular dispersion *w*_χ_ and D) fibril diameter 2R across the BCU. Left: superficial zone; Right: calcified plate. Filled symbols: averaged within each loading rate group with *n* = 3 in each group (error bars: standard deviations); open symbols: individual samples. Green: low strain‐rate; blue: medium strain rate; red: high strain rate. Horizontal rectangles: guides to the eye; anatomical zone separations (SZ: Superficial; TZ: Transitional; DZ: Deep; CP: Calcified plate). Binning and averaging done prior to percentage calculation to avoid mixing data from different tissue types. All percentage strain were scaled to eliminate errors arising from different levels of tissue strain reached (Experimental Section).

To enable such microscopic resolution of the fibrillar mechanics, point‐to‐point comparisons of ECM nanostructural changes were calculated by normalizing the articular cartilage and calcified plate thicknesses to a scale of 0–100 and calculating binned values, nonlinear trends, and percentage changes on the data (Figure S2, Supporting Information). Since the principal ECM variability is along the vertical surface‐to‐bone axis, tissue depth was normalized as a fraction of distance to the calcified plate from the surface, and SAXS scan‐points at the same depth laterally averaged. The results show (absolute: **Figure** **4**; percentage change: **Figure** **5**) that a) ultrastructural metrics like radius, strain variability, alignment, and fibrillar strain (from the D‐period changes) show significant changes across both anatomical and loading axes, and b) the *relative* size of the changes differs across metrics by a factor of >100. Fibril D‐period continuously increases (in unstressed state) across the cartilage thickness and decreases in the calcified plate (Figure 4A). The reasons for this variation likely include increased hydration (due to increased proteoglycan content) in going from the superficial to deep zone in articular cartilage, and biochemical differences between type I (subchondral bone in lower calcified plate) and type II (articular cartilage and calcified cartilage in upper calcified plate) may play a role. Compressive loading reduces the D‐period not only in the articular cartilage, but also in the initial part of the calcified plate as well, as indicated by the small arrow in Figure 5A (an unexpected result given the high stiffness ≈10–20 GPa of this layer^[^ ^20^ ^]^). Right at the superficial zone, D‐period is enhanced by ≈0.2% on loading (arrow), but on going further through the transitional and deep zone, the D‐period change goes through 0% (in the transitional zone) to ≈−0.2% at the bottom of the deep zone; a continuous gradient indicated by the grey triangle in Figure 5A. The angle of the fibrils in the superficial zone is at 90° to those of the deep zone fibrils. The increase in D‐period at the superficial zone likely arises from a Poisson type lateral expansion of the cartilage upon compression, due to which fibrils in the plane of the surface will elongate perpendicular to the deformation direction. This mechanism is quite distinct from the reduction in D‐period in the deep zone, likely due to the loss of swelling pressure due to hydration loss in the glycosaminoglycan network. To quantitatively test whether these spatial‐ and load‐induced variations in ECM nanostructure are significant, generalized additive models (GAMs) were also used to model the variation, and the results showed highly significant (*p* < 0.001) differences with both anatomical location and loading state (Table S2, Supporting Information). In Figure 5, the initial loading strain rate did not significantly change the percentage changes, with the one exception of the D‐period, where the highest strain‐rate D‐period was higher than the medium‐ and low‐rate cases (Table S3, Supporting Information).

Fibril diameter, strain variability and alignment, however, show entirely different trends which are much more responsive to load‐induced changes (Figure 4A,B) and much larger in magnitude. In the unstressed tissue, fibril diameter increases from superficial to the deep zone (Figure 4D), within the reported range^[^ ^14^ ^]^ of ≈60–70 nm for type II collagen fibrils. However, compression (Figure 5D) induces a compressive change of ≈5–10% across most of the tissue, a factor of 10–20× larger than relative changes in D‐period (linked to fibril strain). This finding provides clear circumstantial evidence that load‐induced water diffusion, both from within the fibril to outside (fibril diameter) and out of GAGs in the extrafibrillar space, mediate mechanical changes in cartilage tissue.

The rapid increase in fibril diameter across the calcified plate (Figure 4D) from ≈60 to 120 nm is likely reflective of the change from Type II collagen fibrils to Type I (≈100–200 nm^[^ ^21^ ^]^). Statistical variability (from error bars) is much greater in the calcified plate, likely due to a combination of (a) partial convolution of scattering signals from calcified cartilage, subchondral bone, and articular cartilage due to sample irregularity and (b) larger inherent variability in all fibrillar parameters due to the mineralization‐induced disordering of collagen molecules.^[^ ^22^ ^]^ Nevertheless, under compressive loading we observe a slight increase in fibril diameter in the calcified region (angled small arrow, Figure 5D right). This discontinuity in behavior may be linked to vertically oriented fibrils in the calcified plate^[^ ^23^ ^]^ undergoing a Poisson‐type expansion on being compressed vertically. The complementary small axial contraction (D‐period; Figures 4 and 5A) is consistent with this behavior and highlights the different mechanical response in articular‐ versus calcified cartilage regions. In articular cartilage fibrils both lose axial strain and compress laterally, as expected from a partially water‐filled fibrous hydrogel, while in calcified cartilage, the behavior is like an aligned hard fiber composite.^[^ ^24^ ^]^ Since fibrils are continuous across the cartilage‐calcified cartilage interface, these ultrastructural differences will result in significant small‐scale (microscopic) strain gradients. Exploring the mechanical implications and ultrastructural origins of this discontinuity, as revealed by these measurements, would be an important future area of study.

Fibril strain variability and alignment (Figures 4 and 5B,C) characterize the behavior of an aggregate of fibrils rather than a single fibril and are thus complementary to D‐period and diameter, which report average characteristics of the individual fibril. In unstressed tissue (Figure 4B), strain variability is maximal in the superficial zone, reduces smoothly across the transitional‐ and is nearly constant in the deep zone. Loading (Figure 5B) increases the strain variability by up to 30–50% (a 250×‐fold increase compared to the 0.2% shifts for D‐period and 10% for diameter), with the main changes occurring in the superficial and transitional zones (horizontal brace; Figure 5B). For fibril alignment, increase in w_χ_ on loading (Figure 5C) shows that fibrillar alignment decreases on loading in articular cartilage. The effect is most pronounced in the transitional zone where randomly oriented fibers can be easily pushed into other orientations, and least in the superficial zone. These changes are as expected for a fiber composite: since superficial zone fibrils are oriented perpendicular to load, they will remain aligned on compression, and transitional and deep zone fibrils will splay out conically^[^ ^25^ ^]^ when compressed, increasing w_χ_. For both fibril strain variability and alignment, the deep zone/calcified plate interface acts as an “anchor” where the load‐induced changes go to zero (Figure 5B,C, overlapping lines on right hand sides). When compared to the D‐period (linked to fibril strain) the order of magnitude larger % increases in fibril strain variability, orientation, and fibril diameter under compression indicates the need for bespoke measures and models of mechanical response at the nanoscale for soft ECM‐rich tissues, going beyond fibril axial strain and traditional fiber‐composite models.

### 2.5 From Fibril to Molecular: Transitions in Molecular Order, Packing, and Crystallinity

The foregoing observations challenge us to explore further, at a fundamental level, a) the intrafibrillar (molecular) mechanisms driving these zone‐specific changes in the fibrillar mechanical response across the BCU and b) how these molecular mechanisms contribute to the large tissue strains (≈30%), when individual fibril strain levels are <1% (Figure 5).

**Figure 6:**
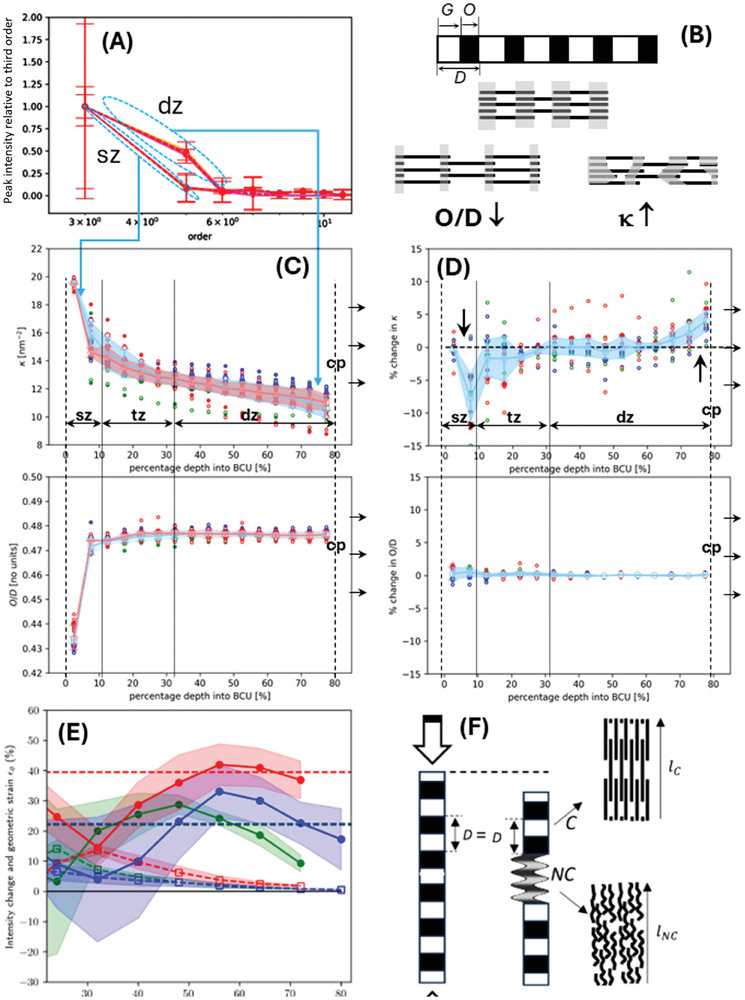
Intrafibrillar disorder, lateral molecular shifts and crystalline‐noncrystalline fibrillar transitions enabling strain: A) Peak intensity ratios I_n_/I_3_ for superficial (sz; left) and deep (dz; right) regions, with *n* = 3,5,6,7,8,9,10, and 11. B) Schematic of intrafibrillar changes in lateral shift of tropocollagen molecules (changing O/D; left) and disorder at the gap/overlap interface (changing disorder parameter κ, right). C) Variation of O/D and κ across the depth of articular cartilage; data is binned data per sample color‐coded by strain rate: Green: low; blue: medium; red: high D) Corresponding percentage changes in κ and O/D; Arrows: changes in κ at superficial/transitional and bone‐cartilage interface. E) Load‐induced reduction (in absolute value) in X‐ray collagen peak intensity I_0_ (filled circles) and geometric fibril strain (open symbols), as a function of % depth in the BCU; data points are binned averages (*n* = 3 per strain rate); shaded regions denotes ± standard deviation. low‐ (green), medium‐ (blue), and high‐ (red) loading strain rates F) schematic of the fibrillar kinking model: left: blurred shading, kinked segments, and a molecular level view (right), with segments denoting tropocollagen molecules.

For a), we go beyond the single‐order peak (*n* = 3) analysis, to analyze the zonal‐ and load‐induced variation in peak intensities across multiple diffraction orders *n* (Figure S9, Supporting Information). From diffraction theory, the meridional SAXS peak intensity of a single fibril depends on the axial intrafibrillar organization of tropocollagen molecules.^[^ ^26^, ^27^, ^28^ ^]^ As shown in **Figure** **6**B, the intrafibrillar organization is not perfectly regular; axial shifts of molecules relative to their neighbors lead to internal disordering/blurring of the gap/overlap interface. Further, changes in the relative lengths of the gap/overlap regions will alter peak intensities in an order‐ and electron‐density profile‐dependent manner. These changes will also have biochemical and biological implications, by changing which collagen side‐groups are exposed on the surface to other ECM molecular components for binding and signaling. A simplified diffraction model for the peak intensity ratios can be developed (Section S4 end, Supporting Information) in terms of the intrafibrillar disorder parameter (κ), which measures the fuzziness of the gap‐overlap interface and the overlap/D‐period ratio (O/D).

Applying this model to the data (Figure 6C) reveals 1) the intrafibrillar disorder parameter κ is much higher in the superficial and early transitional zone compared to the deep zone and 2) the gap/overlap ratio O/D (reflecting molecular arrangement and stacking) likewise exhibits a transition from low to high O/D over the same region. Figure 6A shows that the intensity‐ratios/order plots of the superficial versus deep zones are clearly different, with a more rapid fall‐off of peak intensity with order *n* in the superficial zone indicating higher intrafibrillar disorder κ. Further, on progressing toward the bone‐cartilage interface, there is a continuous decrease of disorder (mirroring the gradient in axial fibril strain seen in Figure 5A) and a parallel increase in O/D toward 0.5. The results reveal that the superficial zone has a distinct intrafibrillar organization from the rest – greater disordering of the gap/overlap transition and intrafibrillar molecular shifts (Figure 6B). This difference may play a role, together with orientation differences (Figure 1A), in localizing the stress response to injury in the superficial zone ECM and modulating zonal changes in fibrillar‐level mechanics.

Under loading, we see a local decrease in intrafibrillar disorder at the superficial‐transitional interface and an increase right near the calcified interface. These findings are robust to removing the high‐strain rate group (with higher strains and consequently more disorder) from the analysis. The gap‐overlap ratio shows little load‐induced change over the same scale. Thus, we have identified a change in the regularity of the gap/overlap interface as one molecular‐level structural mechanism at these two regions during compression.

Considering the different modes of strain now, these can include fibril reorientation, axial contraction, and interfibrillar shearing. The reorientation‐dependent strain can be calculated from the change in the FWHM of the angular distribution *w*(θ; Δθ) upon loading $\varepsilon_{G}=\frac{\cos{\left\langle \theta \right\rangle }_{\Delta \theta (\varepsilon )}-\cos{\left\langle \theta \right\rangle }_{\Delta \theta (0)}}{\cos{\left\langle \theta \right\rangle }_{\Delta \theta (0)}}$. The dashed lines in Figure 6A show that geometrical (reorientation) strain is larger in the transitional zone and decreases from ≈10–20% to near zero in the deep zone, while axial contraction (fibril D‐period changes) is much smaller (<1%) than applied strain throughout. In the region between 40–70%, scattering power *I*_0_ decreases considerably (up to 20–30%) and is proportional to 1) number of crystalline fibrils in the scattering volume, 2) electron‐density difference between gap and overlap zones 3) the intrafibrillar disorder and O/D (diffraction model above) and 4) out‐of‐plane tilts. If changes in 2) are operative, then the density difference reduces between the gap and denser overlap regions on compression, which is hard to envisage. For 3), Figure 6D shows that κ and O/D do not change in the deep zone, which appears to rule that out. Hence, we consider mechanisms 1) and 4). Since fibrils are kept straight and aligned by the swelling pressure of the hydrated PG‐rich extrafibrillar gel and intrafibrillar water,^[^ ^18^ ^]^ upon compression, the loss of fluid from both these phases will reduce stress on the fibrils, and a fraction *f*_NC_<1 of the axial gap/overlap segments may collapse and undergo internal kinking, like a deflated tyre (Figure 6C). This paracrystalline disordering^[^ ^29^ ^]^ of the periodically arranged tropocollagen molecules is driven by partial fibrillar‐level dehydration and lateral compaction. The calculations for the disorder parameter κ earlier refer to the remaining crystalline fibrils only, so this is a separate effect. The kinking of the fraction *f*_NC_ causes a shortening of the total length of fibrils upon compression and contributes to tissue strain. A model (Section S3, Supporting Information) combines experimental changes in I_0_ and fibril diameters with the dehydrated/hydrated intermolecular spacing to predict tissue strain. These are shown as dotted lines in Figure S10 (Supporting Information), corresponding to ≈39% of the fibrils per unit volume axially kinking, resulting in a reduction of length by 60%. This behavior of the hydrated fibrillar gel in AC is distinct from elastic deformation of fibers in a solid matrix, seen, for example in collagen fibrils in a mineralized extrafibrillar matrix in bone.^[^ ^30^, ^31^ ^]^

However, it is possible that these numbers are overestimates, since out‐of‐plane tilting of the fibril distribution (Figure 2) also contribute to apparent reductions in I_0_ (and consequent peak intensity reductions). To test this, the 2D SAXS pattern changes on going from an initial in‐plane 3D conical distribution to an out‐of‐plane tilt of 20° (Figure S9B and Section S6, Supporting Information) were simulated (combined with the measured ≈30% increase in angular width w_χ_ upon loading; Figures 4 and 5) and compared to the reduction in experimental meridional peak intensity (Figure S9A, Supporting Information) for a range of final tilt values. For a pure in‐plane splaying and reduction of *I*_0_, the angular position will not change, but the intensity will reduce, while for a tilt‐induced reduction, shifts in both angular position and intensity occur. We find (Figure S9B, Supporting Information) that experimental shifts in these parameters lie between the downward‐only shift and the predicted curves for out‐of‐plane tilt, indicating that both mechanisms (fibrillar kinking and out‐of‐plane rotation) are likely operative.

In the fibril kinking scenario, while spatial and load‐driven changes in the D‐period in collagen fibrils in AC arise due to a combination of thermodynamic and elastic strains, as noted earlier, we can obtain an approximate estimate of the absolute collagen strain in the deep zone. If the thermodynamic eigenstrain is taken as constant in the deep zone, upon compression, the reduction in D‐period is due to elastic strain, which leads to fibrils reaching their unstressed (reference) strain (minimum D‐period) just at the onset of disordering and kinking. From Figure 4A, the D‐period in the deep zone in the compressed and uncompressed state is ≈66.8 and 67.0 nm, respectively. In this scenario, the deep‐zone fibrils are in a positive absolute strain state of ≈0.3% in the uncompressed cartilage. However, since 1) the eigenstrain can vary across the tissue (changing the internal reference), and 2) the internal kinking mechanism is mainly operative in the deep zone (Figure 6E) making it hard to confirm whether this reference was reached everywhere on loading, this approach cannot be used to calculate absolute strain across all parts of the tissue.

### 2.6 Nonlinear Coupled ECM Deformation Mechanisms Across Multiple Modes

**Figure 7:**
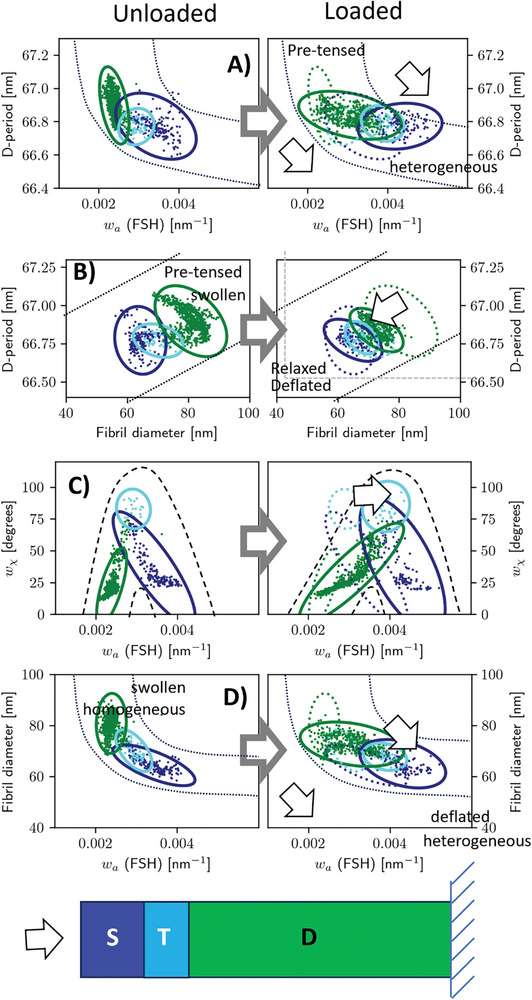
Correlation across the modes shows evidence of a nonlinear structural response: Correlation plots with confidence ellipses for D‐period, angular and axial strain heterogeneity, and fibril diameter. Left: unloaded state; right: loaded state with confidence ellipses from unloaded state retained to indicate direction of change. Dark blue: superficial; light blue: transitional; Green: deep zones. Dotted lines and arrows are guides to the eye only; text annotations are to provide context. Data points are from all samples (*n* = 3 samples/strain rate; 3 strain rates).

**Figure 8:**
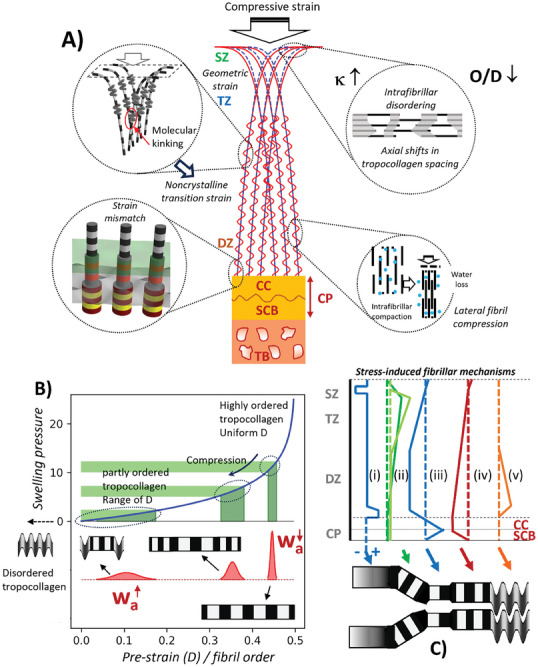
Model of the multiscale fibrillar deformation modes across the BCU: A) Middle: Schematic of the fiber arcades in articular cartilage prior to (blue) and after (red) deformation; Insets (clockwise top right): 1) superficial zone disordering 2) lateral fibril compression 3) strain mismatch at calcified interface and 4) fibrillar kinking in deep zone. B) Fibrils across the BCU exist in a range of states of pre‐strain and strain heterogeneity. In the deep zone (right), fibrils are highly aligned and pre‐stressed; narrow distribution width in D. Red curves – distribution of D‐period linked to w_a_. Change of swelling pressure (across the tissue and during compression) causes nonlinear changes in fibril strain, heterogeneity and kinking. C) Schematic gradients of load‐induced changes in i) intrafibrillar disorder κ ii) angular dispersion w_χ_ and strain heterogeneity w_a_ iii) diameter 2R iv) axial D‐period and v) molecular‐level kinking; fibrillar schematic at bottom shows what the structural changes due to i)‐v) represent physically. SZ: superficial zone; TZ: transitional zone; DZ: deep zone; CP: calcified plate.

The cartilage mechano‐structural response is not a single/discrete change, but lies on a continuum across the tissue, where different supramolecular deformation modes interact with each other differently in distinct tissue zones. Considering the articular cartilage only (excluding the calcified plate), **Figure** **7** shows a color‐coded representation of how the distinct fibrillar modes (D‐period, w_χ_, w_a_, and 2R) correlate with each other across the tissue thickness, with ellipsoidal regions denoting clusters for each zone (Section S2, Supporting Information). The axial D‐period and strain‐heterogeneity are oppositely correlated in a nonlinear manner (Figure 7A) – the most highly pre‐strained regions (from changes in D‐period; in the deep zone) have the least variability in D‐period. Further, on compression, the *average* D‐period in fibrils reaches an asymptotic value of ≈66.7 nm, but – especially in the superficial and transitional zone – the variability of the D‐period increases substantially. This data suggests that cartilage fibrils can have low D‐period bands (lower pre‐strain) along the fibrils (leading to strain broadening, increased strain heterogeneity in w_a_), which – like a set of disordered hydrophilic fibers absorbing water and getting aligned and stretched – can increase their D‐period and align at the same time (**Figure** **8**B).

The positive correlation between fibril D‐period and fibril diameter (Figure 7B) in the unloaded state supports the internally hydrated picture of the collagen fibril – water molecules within the fibril increase fibril diameter by increasing lateral tropocollagen molecule spacing, and the high hydration (both internal and external) increases the strain along the fibril, like inflating a sponge. During compression the axial versus radial strain (Figure 7B) shows the “deflating sponge” picture most clearly – compression shifts both measures toward asymptotic values of 66.7 and 60 nm. The relative magnitude of the shift provides a measure of the hard‐to‐access direct stiffness tensor of the fibril. Specifically, if the axial stiffness is E_F(ax)_ and the transverse is E_F(tr)_, and the hydrostatic swelling pressure is assumed to be isotropic, then the axial and transverse strains are measures of the respective compliances (inverse stiffness), and the slope of the guideline ≈0.02 is ≈$\frac{E_{F(tr)}}{E_{F(ax)}}\frac{D}{R}$ where D≈66.8 and R≈70 nm are average values for the D‐stagger and fibril diameter. Using E_F(ax)_≈1–2 GPa from single fibril tensile measurements,^[^ ^32^ ^]^ E_F(tr)_ is ≈20–40 MPa, which is, gratifyingly, in the range of previous scanning probe microscopy measurements of laterally deposited fibrils on substrates,^[^ ^33^ ^]^ and measured in situ in “in vivo like” microenvironments.

Axial strain heterogeneity versus fibril misalignment (Figure 7C) shows that fibrils in the transitional zone mainly increase their strain heterogeneity on compression, probably because their degree of misalignment is already high (high w_χ_). Superficial zone fibrils show a negative correlation between misalignment and strain heterogeneity – the most aligned fibrils (right at the superficial zone) have the greatest axial strain heterogeneity. Compression sends these superficial fibrils into a state of greater alignment and strain heterogeneity; potentially, some of these high‐strain zones may be key weak points causing fissuring or damage at early‐stage osteoarthritis. Conversely, deep‐zone fibrils show a positive correlation between misalignment and strain heterogeneity; fibrils toward the upper end of the deep‐zone (≈20–40% normalized BCU distance; Figure 5) show both increased strain heterogeneity and reduced alignment, but this decreases toward the bone‐cartilage interface. However (Figure S5B, Supporting Information), D‐period and angular misalignment in the deep zone are oppositely correlated – fibrils in the upper part (“*u*” label: Figure S5B, Supporting Information) of the deep zone are more misaligned to start with, and hence show greater reorientation to larger w_χ_ on compression, while fibrils in the lower part (“*l*” label: Figure S5B, Supporting Information), closer to the anchoring calcified cartilage, do not have this flexibility to reorient, and must therefore accommodate compression by reduction of axial D‐period and molecular kinking as described above. This can also be seen in the change in scattering intensity I_0_ on compression (Figure S5A, Supporting Information), showing that it primarily occurs in the deep zone. Finally, the negative correlation between fibrillar diameter and strain‐heterogeneity in the native state (Figure 7D), and clear shift toward lower diameters and increased heterogeneity on compression, is consistent with a hydration‐driven exudation of fibrils during compression, which may occur heterogeneously along the fibril length leading to strain broadening.

Comparing the articular cartilage to the calcified plate (a mixture of calcified cartilage and subchondral bone), in Figure S6 (Supporting Information), further clarifies the distinct modes of deformation across the mineralized/unmineralized interface in the BCU. Figure S6A (Supporting Information) shows that fibrillar D‐period has a much wider variation in the calcified plate, but reduces on average upon compression, with little change in strain heterogeneity. Likewise, fibril diameter decreases upon compression, again with little change in strain heterogeneity.

## 3 Summary and Conclusion

These mechano‐structural insights are integrated schematically in Figure 8, which is centerd around the Bouligand‐type graded fibril orientation and expands to identify distinct fibril deformation dynamics across anatomic zones (inset bubbles). Considering the zonal average (from Figure 7), superficial zone fibrils get more disordered both axially (w_a_) and angularly (w_χ_), but average D‐period doesn't change much. However, microbeam mapping identifies elevated fibril strain in the uppermost part of the superficial zone. As this region is in contact with the adjoining joint, the elevated strains and stresses may be linked to superficial zone fissuring observed in injury and OA.^[^ ^34^ ^]^ The altered intrafibrillar disorder and gap/overlap ratio in superficial zone fibrils may be a factor in enabling the elevated strain (upper right inset in Figure 8A). Just below the superficial zone, the deformation is accommodated by what we call geometric strain, i.e., reorientation of fibrils (Figure 6A, dashed lines), consistent also with earlier work by Moger and colleagues^[^ ^16^ ^]^ and recent phase‐contrast CT work.^[^ ^13^ ^]^ However, where fibrils are more aligned in the deep zone there is a transition to the molecular kinking mechanism, possibly combined with out of plane tilting of the distribution. Changes to topography of the calcified interface (as may occur in osteoarthritic driven remodeling) would significantly change the anchoring of the fibrils and alter the spatial dependence of the fibrillar deformation and intra‐ to interfibrillar water flow (among other factors). The increase in fibril‐strain toward the calcified interface is likely related – if significant angular broadening is restricted, fibrils find other ways to accommodate macroscopic strain, either kinking along their length or contracting, likely driven by fluid‐flow.

From transitional to deep zone, the crystalline fibrillar phase exhibits, on compression, a nonlinear reduction of D‐period coupled with increased strain heterogeneity, a phenomenon shown in Figure 8B, where swelling pressure from proteoglycans (which acts to pre‐tense fibrils^[^ ^28^ ^]^) reduces on compression due to water‐loss. The schematic integrates the experimentally observed variable D‐period, changes in intrafibrillar order and organization, increases in strain heterogeneity, and local loss of crystallinity along the fibril, and the directional shift on loading (curved arrow to left). It envisages a fibrillar network with variable pre‐strain (shift in D‐period distribution in Figure 8B) and increased strain heterogeneity (increased w_a_) and partially kinked axial patches (reduced intensity). Increased swelling pressure acts to align this fiber network. However, as the segments with reduced or variable D‐period (or kinked) are distributed randomly along the fibrils in the scattering volume, the link between stress and average D‐period is non‐linear – first, limited increase in stress as most of the fibril segments aren't aligned, and later a rapid increase when the fibril segments have aligned, like J‐shaped stress‐strain curves for tissues like skin in tension. When the tissue is compressed, the loss of water from both extra‐^[^ ^28^ ^]^ and intra‐fibrillar compartments (Figures 5D and 7B,D) cause a loss of swelling pressure, shifting the average D‐period down and to the left non‐linearly, consistent with the cluster analysis in Figure 7. Underlying molecular‐level mechanisms may involve processes like load‐induced reorientation of tropocollagen molecules (proposed earlier for cornea experimentally^[^ ^19^ ^]^ andtheoretically^[^ ^35^ ^]^).

Going between deep‐zone AC fibrils and the calcified cartilage, Figure 8A (lower inset) shows the pre‐ and post‐loaded states, changing from contracted, deflated fibrils in the AC to contracted by laterally Poisson‐expanded fibrils in the upper part of the CP (possibly in the calcified cartilage component). Strain and stress heterogeneity could also play a role in the increased fuzziness of the gap‐overlap interface (via κ) in uncalcified fibrils just before the interface. It is notable that D‐period changes at low‐stress levels (<1 MPa) cause reductions in fibril strain in the upper part of the calcified plate (which is likely calcified cartilage) but drop to zero shortly after; differences in fibrillar mineralization between Type II (calcified cartilage) and type I collagen (subchondral bone) may play a role.

The findings have relevance in the mechanobiology of osteoarthritis, injury‐induced and age‐related degeneration. In ageing, matrix composition ratios change (e.g., proteoglycan content decreases), collagen cross‐linking by age‐related glycation increases, and in post‐traumatic osteoarthritis, the morphology of the calcified tissue interface is altered along with changes in nanostructure^[^ ^12^ ^]^ and underlying bone mineralization and structure.^[^ ^36^ ^]^ The challenge is to understand how global mechanical stimuli interact with these changes to modify the local (cell/tissue) and organ‐level biomechanics and disease progression. The SAXS‐mechanics imaging methodology has demonstrated the ability to quantify these mechanisms in detail at the nano‐ and micro‐scale, in physiologically realistic tissue constructs incorporating both cartilage and bone, demonstrating feasibility for studying age‐related changes and injury.

Our study has several limitations. We assume that D‐period shifts of the fibrils in each microscopic scattering volume are similar; inclusion of a parameterized angular dependence is possible in our model for future work. Imaging a 3D fibrillar structure with 2D scanning (even with 3D modelling of the scattering) requires the assumption of fibril symmetry around the vertical axis. While the coring protocol ensures that sample geometry is in the form of Figure 8A, and average fibril angle (in the deep zone) is consistently along 90° in the I(χ) plot, with little change pre‐ and post‐deformation (Figure S3, Supporting Information), it is possible that the main fibril axis is slightly out of plane. However, as shown in the model simulations (Figure S9, Supporting Information) it can be quantified how much of the intensity change might be affected by out‐of‐plane tilt. Tensor tomography methods^[^ ^37^, ^38^, ^39^ ^]^ combined with loading will circumvent this limitation although taking longer. The nominal grip strain applied (30%) is higher than typical physiological strains (20%); however, the actual strain on the articular cartilage is ≈20% for two of the three loading rates used, and the fibrillar‐level phenomena observed do not show qualitative changes between these and the highest strain rate. Strain‐control (not load control) means the deformation state is not fully physiological in terms of load. Biomechanical forces are considered statically, which is an inherent feature of the scanning SAXS approach; localized dynamic measurements focusing on a single anatomical location^[^ ^25^ ^]^ will be useful complements. Though sharp increases (in diffuse SAXS scattering) and decreases (collagen peak intensity) are visible at the boundary to the calcified plate (Figure 2C,D), potential 3D corrugation or waviness of the boundary along the beam direction could lead to signals from both components mixing right at the interface; full‐field SAXS tensor tomography methods would overcome this limitation. The matrix of cartilage contains several other important components (proteoglycans,^[^ ^40^ ^]^ elastin^[^ ^41^ ^]^ etc.) which can only be indirectly analyzed here, by their effects on the fibrillar network. While anticipated effects of radiation damage are well below the magnitudes of induced changes in the SAXS patterns (Figure S7, Supporting Information), reducing exposure times still further (e.g., to ≈100 ms) will help, albeit at the cost of worse count statistics. The clearest gap is the lack of concurrent microstructural imaging to complement the nanoscale structure (e.g., using phase‐contrast X‐ray imaging reported recently^[^ ^13^ ^]^), with tomographic methods^[^ ^42^ ^]^ having shown promise in detecting residual microscale strains in the calcified interface,^[^ ^8^, ^43^ ^]^ and combination of both methods would provide much greater insights.

In summary, a 3D scattering and imaging methodology was developed to deconvolve the complex, graded deformation mechanisms in the fibrillar network in the intact bone‐cartilage unit, which led to the discovery of mechanisms both arising from and feeding back to higher‐level structural organization. Notably, we find that the dominant deformation mechanisms are not the hitherto standard shifts in axial periodicity (D‐period) but much larger changes in fibril diameter, crystallinity, strain heterogeneity and angular reorientation and that these changes vary in a coupled, nonlinear, and correlated manner across the bone‐cartilage unit. At the surface, the collagen molecular structure was distinct from the bulk, with increased intrafibrillar disorder and reduced axial tropocollagen overlap, and compression locally reduces disorder near the surface but elevates at the bone‐cartilage interface. A molecular kinking associated with a crystalline to noncrystalline transition, coupled with out‐of‐plane tilting, was identified as the enabling mechanism for tissue strain in the deep zone, versus an orientational mechanism toward the surface. Across the hard(calcified)/soft(articular cartilage) interface, a qualitative switch between fibrillar deflation and compaction in the articular cartilage and hard‐composite Poisson expansion in the connecting fibrillar network may result in significant small‐scale mechanical stresses, which could be exacerbated in post‐traumatic OA.

## 4 Experimental Section

### Sample Preparation

Bovine BCU explants (2 mm diameter x 5 mm length) were cored out of metacarpophalangeal (MCP) joints freshly obtained from a commercial abattoir (C Humphreys & Sons‐ Blixes Farm Shop, 142 Chelmsford, UK), under constant irrigation to minimize frictional heating,^[^ ^17^ ^]^ inspired by a protocol by Burgin and Aspden.^[^ ^44^ ^]^ The custom‐built coring rig had tilt controls to enable the explants to be machined out normal to the joint surface at each point. Cores were placed in Eppendorf tubes with Dulbecco's Modified Eagle's Medium – low glucose (DMEM) (Sigma‐Aldrich, Poole, UK), and flash frozen till the synchrotron SAXS tests.

### In Situ Micromechanical Testing

A waterproof 3D printed chamber with Kapton windows was attached to a microcompression rig (44 N load cell rating) used previously^[^ ^6^ ^]^ and controlled via a custom LabVIEW interface allowing strain‐ and strain‐rate controlled experiments, reported previously.^[^ ^6^, ^28^ ^]^ The rig was mounted in the I22 SAXS/WAXS beamline at Diamond Light Source (DLS), for in situ SAXS experiments during stepwise compressive loading. Cores were thawed just prior to the experiment and mounted in the rig, with the chamber filled with DMEM to keep the tissue physiologically hydrated during testing and SAXS scans.

Uniaxial unconfined compressive stress‐relaxation tests were carried out with synchrotron SAXS scanning. After tare loading to 0.1N, a rectangular 2D SAXS area scan was performed on the BCU cores, with the long axis along the depth from the surface at this position (unloaded scan). AC thickness was found on a per‐sample basis from the X‐ray transmission data, from which the load‐platen displacement was calculated for a nominal 30% (grip) compressive strain. The exact strain applied was calculated after each experiment from the mapping data as detailed below. Three different loading strain rates were used (0.22%, 10%, and 100%.s^−1^) with three sample repeats per strain rate (i.e., *n* = 3 per strain rate and total 9 samples). Due to a technical issue with the LabVIEW control software at high motor speeds, the high strain‐rate displacements were larger than target values, and the excess could be quantified. After compression, a hold period of 300 s was used for viscoelastic relaxation, followed by a 2D SAXS area scan (loaded scan).

SAXS measurements used a microfocus X‐ray beam size 20 µm diameter at 14 keV and a sample‐to‐detector distance of 5.8 m with a Pilatus 2 m detector^[^ ^45^ ^]^ (Dectris, Villingen, CH) (pixel size 172 µm; resolution 1475 × 1679 pixels). SAXS scans were in fly‐scan mode (1.0s/scan point) for rapid data collection and minimizing the radiation‐induced structural damage. 2D area scans (2.6 mm height; 0.8 mm width) were performed with 40 µm step‐size (2x beam diameter) across the BCU, which included the full AC and CP tissue but only a part (≈1.4 mm) of the ≈4 mm long TB region.

### Radiation Damage

Excessive exposure to high‐intensity synchrotron X‐ray beam could cause damage to the extracellular matrix of biological tissues,^[^ ^46^, ^47^ ^]^ disrupting the fibrillar ordered structure. This leads to a reduction in meridional peak intensity, increased axial width, and reduced D‐period. To check the radiation damage threshold was not exceeded, 1) the total radiation dose *D* was calculated like the process in^[^ ^46^ ^]^ and 2) carried out repeat exposures of the tissue to the X‐ray beam to check that the exposure protocol was below the level at which damage starts to occur. The radiation dose/point per scan was 12 kGy, and total radiation exposure/point between 12–24 kGy, with an average of 18 kGy over two 1s exposures, with a wait time of 60 min between exposures (time between consecutive SAXS scans). Each tissue location was exposed between 1x and 2x due to sample displacement between scans (11 kGy s^−1^ during the exposure). In 2), repeat exposures of 1s with no wait time showed ≈5% reduction in peak area after 3x exposures, by which point a radiation dose of 36 kGy would had been delivered at the tissue location. This was consistent with the radiation damage threshold of 35 kGy seen in bone,^[^ ^47^ ^]^ and with prior work on Type I collagen in tendon where 6–7 kGy s^−1^ showed little radiation damage to the collagen ultrastructure, but higher doses (106–124 kGy s^−1^) showed significant reductions in area and increase in peak width.^[^ ^46^ ^]^ Hence the radiation damage threshold was not expected to had been exceeded in these experiments. Details are given^[^ ^46^, ^47^ ^]^ Section S5 (Supporting Information).

### Data Reduction

2D SAXS patterns were reduced to radial (I(q)) and azimuthal (I(χ)) profiles using DAWN (www.dawnsci.org)^[^ ^48^ ^]^ as described earlier.^[^ ^6^ ^]^ Intensity profiles were transmission‐corrected using the transmitted beam diode intensity at the detector. From radial profiles, the integrated area under the 3rd‐order meridional peak, with (total) and without (collagen‐only) diffuse SAXS background, was calculated. As an estimation of fibril direction (in the 2D plane), a Gaussian model was fit to the azimuthal I(χ) profile. The combination of total and collagen‐SAXS intensity along with fibril direction was used to classify the scan points into superficial (SZ), transitional (TZ), deep (DZ), calcified plate (CP), and trabecular bone (TB) zones, as described previously for static scans.^[^ ^17^ ^]^

### 3D Diffraction Model

The meridional scattering from a single fibril (Figure 1) was modeled in reciprocal space as a series of intensity ellipsoids, spaced 2π/D apart, where D is the meridional stagger and a marker of axial (pre‐)strain.^[^ ^28^ ^]^ The width *w*_p_ in the flat direction is inversely proportional to the fibril radius R, while width along the narrow direction *w*_a_ is proportional to the axial pre‐strain heterogeneity as *Dq*_0_ *w_a_* =  Δ*D*. For each scan point, the full SAXS signal is an integral over the scattering from single fibrils, oriented in 3D space, and the measured intensity is the 2D intersection of the 3D reciprocal space scattering with the Ewald sphere. To calculate the measured SAXS intensity, the scattering from a single fibril was convolved with 3D fibril orientation distributions *w*(θ, ϕ) on the Ewald intersection surface, where the distributions depend on the zone identified above. Full details are in Section S1 (Supporting Information), and simulations of the model are shown in Figure 2.

Experimental *I*(*q*) and *I*(*χ*) data were fit to models of the radial and azimuthal integrals of the resultant convolved intensity, using custom Python code (Anaconda Continuum) with the *CuPy* package^[^ ^49^ ^]^ for GPU‐accelerated fits of the spherical integration on a desktop PC (i5 Intel, Nvidia GeForce GTX 1050Ti 4 MB GPU). Fitted fibrillar parameters were 1) D‐period 2) strain heterogeneity w_a_ 3) diameter 2R 4) angular width of fibril distribution (polar angle Δθ) and 5) scaling prefactor I_0_.

### Data Representation and Statistical Analysis—Pre‐Processing

Spatial maps of the fibrillar structural parameters and SAXS metrics (total and collagen intensity) were rendered using the Python package *matplotlib*. From these maps, minor variations in the articular cartilage thickness in the lateral (x‐) dimension of each scan could be observed. Therefore, for an accurate estimation of the tissue strain in each sample, strain was estimated by calculating (at each x‐point) the fractional change in length of vertical lines drawn from the top of the superficial zone to the start of the calcified plate, and the average was taken as the tissue strain per sample; tissue‐level deformation in the calcified plate was ignored as it was below the SAXS‐step resolution (40 microns). To enable a comparison across samples, a scaled depth‐axis was calculated with 0–80% in the articular cartilage and 81–100% in the calcified plate (the trabecular bone was excluded from analysis in the current work), based on average relative lengths of articular cartilage and calcified plate.

### Data Representation and Statistical Analysis—Data presentation

Within each zone, averages, standard deviations, and kernel density histograms of the fitted parameters were calculated (Figures S1 and S3B, Supporting Information). Subsequently, across the entire tissue using the package *mgcv* in R (R version 4.3.0; RStudio 2023.6), generalized additive models (GAMs) were used to identify spatial trends in fibrillar structural parameters as a function of scaled depth (see Section S4, Supporting Information), as well as load‐induced changes, on a per‐sample basis (*n* = 3 samples at 3 different strain rates, as stated above). Percentage change was calculated by binning in intervals of 10% across scaled depth. For each strain rate (*n* = 3/strain rate), the average of the binned data and percent change was calculated and plotted with standard deviations in Figures 4 and 5 respectively. Since each sample reached different strain levels (Table S4, Supporting Information), the percentage change in each parameter was scaled by (30%/sample strain) for comparability; this eliminates significant deviation otherwise inevitable with the high strain‐rate group. Pairwise correlations between the fitted parameters, on a zone‐specific basis, were plotted with ellipsoidal confidence intervals (Section S2, Supporting Information). For Figure 7 only, for visual clarity, in the pairwise correlations reported, data was treated to remove outliers >3 S.Ds from the mean (see Section S2, Supporting Information).

## Conflict of Interest

The authors declare no conflict of interest.

## Supporting information



Supporting Information

## Data Availability

The data that support the findings of this study are openly available in Queen Mary Research Online (QMRO) at https://doi.org/10.17636/10197845, reference number 97845.
