# Supplementary material for: Nonlinear Stress‐Induced Transformations in Collagen Fibrillar Organization, Disorder and Strain Mechanisms in the Bone‐Cartilage Unit
Source: Adv Sci (Weinh). 2024 Nov 11;12(1):2407649. doi: 10.1002/advs.202407649 (PMC11714194; doi:10.1002/advs.202407649)
Supplement: Supplementary file 1 — Supporting Information [file ADVS-12-2407649-s001.docx]

Supporting Information

**Nonlinear Stress-induced Transformations in Collagen Fibrillar Organisation, Disorder and Strain Mechanisms In The Bone-Cartilage Unit**

*Waqas Badar^1^, Sheetal R. Inamdar^1^, Peter Fratzl^2^, Tim Snow^3^, Nicholas J. Terrill^3^, Martin M. Knight^1^, and Himadri S. Gupta^1,^**

**Section S1: 3D diffraction model**:

The X-ray scattering for a single collagen fibril is related to the form factor for a single cylinder with radius *R* and length *L*, with a periodic electron density along the length with repeat length *D*. The intensity of scattering for a cylinder is proportional to $I(q_{z},q_{\perp})\sim\sin^{2} \left( q_{z}L \right)\left( \frac{2J_{1}\left( q_{\perp}R \right)}{q_{\perp}R} \right)^{2}$ where J_1_ is the Bessel function, q_z_ denotes the reciprocal space vector along the fibril axis, and q_⊥_ is the length of the reciprocal space perpendicular to the axis (the equatorial direction) ^[1]^. The variation in scattering along the axis perpendicular to the long axis of the cylinder (fibril) is given by the second Bessel function term. Since collagenous tissues have a range of fibril diameters rather than a single fixed value, the scattering function was convolved with a normal (Gaussian) distribution of radii, and the intensity profile transverse to the fibril axis was measured and fitted to a Gaussian profile with width *w*_p_. By carrying out the convolution for a range of fibril diameters 2R, the relation between the fibril diameter and 1/*w*_p_ was fitted to a second-degree polynomial, enabling the calculation of fibril diameter 2R for known experimental *w*_p_. Examples of the convolved intensity profile for a range of average fibril radii from 20 to 150 nm are shown in **Figure S9A**, and fibril diameter 1/w_p_ curve is displayed in **Figure S9B** (it is observed the nonlinearity is very slight). From the relation in **Figure S9B**, the fibril diameter can be estimated from the w_p_ parameter.

Due to the axial meridional periodicity *D*, a single fibril will exhibit peaks along q_z_ with spacing 2π/*D*. In 3D reciprocal space, the finite fibril diameter means the axial width of the meridional scattering will be proportional to *w*_p_. We model the total scattering of the n^th^ meridional peak as the sum of a peak term of the type:

$$I\left( q_{z}, q_{\perp} \right)=K_{1}\frac{1}{\sqrt{2\pi}w_{a}}\exp\left( \frac{\left( q_{z}-q_{0n} \right)^{2}}{2w_{a}^{2}} \right)\frac{1}{\sqrt{2\pi}w_{p}}\exp\left( \frac{q_{x}^{2}+q_{y}^{2}}{2w_{p}^{2}} \right)\equiv K_{1}I^{0}\left( q_{z}, q_{\perp} \right)$$

and an empirical nonlinear diffuse SAXS intensity decay, which will be locally subtracted out from the peak analysis. Here *K*_1_ is a pre-factor which is proportional to the electron density contrast between the gap and overlap zones:

$$K={K_{1o}\left( \rho_{O}-\rho_{G} \right)}^{2}$$

and *q*_0n_ = 2π*n*/*D*, with K_1o_ related to detector constants and other experimental configuration parameters. For a fibril oriented in 3D, with, for example, a tilt to the vertical *q*_z_ given by θ and a rotation in the *q*_x_-*q*_y_ plane by φ, the wavevectors are transformed using rotation matrices; we will denote the rotated reciprocal vector as *q*^(θ,φ)^. The unrotated fibril is assumed to be along the *q*_z_ axis (θ=0°).

Since within the scattering volume at each scan point, there are a multitude of fibrils oriented at different angles, the measured scattering will be the sum of scattering from all these fibrils. If the number of crystalline fibril segments in a scattering volume is *N*, and the normalised angular distribution of the fibrils is denoted by *w*(*θ*,*φ*), the total intensity is:

$$I\left( q_{x},q_{y},q_{z} \right)=N\int_{0}^{\frac{\pi}{2}} \sin\theta d\theta\int_{0}^{2\pi} I\left( q_{z}^{\left( \theta,\phi\right)}, q_{\perp}^{\left( \theta,\phi\right)} \right)w\left( \theta,\phi\right)d\phi$$

$$=NK_{1}\int_{0}^{\frac{\pi}{2}} \sin\theta d\theta\int_{0}^{2\pi} I^{0}\left( q_{z}^{\left( \theta,\phi\right)}, q_{\perp}^{\left( \theta,\phi\right)} \right)w\left( \theta,\phi\right)d\phi$$

where $q_{\perp}=\sqrt{q_{x}^{2}+q_{y}^{2}}$ and $\int_{0}^{\frac{\pi}{2}} \sin\theta d\theta\int_{0}^{2\pi} w\left( \theta,\phi\right)d\phi=1$. We denote *NK*_1_ as *I*_0_.

The measured SAXS intensity on the 2D detector is obtained by calculating the 3D reciprocal space vectors which satisfy the Ewald sphere intersection condition and transforming them to detector coordinates, following a standard process ^[2]^. **Figure 2A** shows examples of the 2D patterns. At this stage, intensity reductions due to tilting of the overall distribution away from the Ewald diffraction condition are observed.

For the different tissue zones, distinct angular distributions w(θ,φ) were used with fibre symmetry around θ=0°. Since cores were machined out normal to the joint surface, the fibril orientation distribution was taken as a conical intensity distribution in the DZ and CP zones oriented along the calcified-superficial axis (around θ°=0). In the superficial zone, the orientation distribution was modelled as a 3D belt of intensity in the horizontal plane (near θ=90°), and in the (limited) transitional zone points, as an isotropic 3D intensity distribution with value $\frac{1}{4\pi}$ . These assumptions of projected orientation were verified from experimental measurements of I(χ), with minor in-plane angular deviations corrected for during fitting. For both the superficial and deep zones, the Gaussian width parameter is denoted w_χ_.

In detail, i) deep zone fibrils were represented by a conical (Gaussian) distribution with main direction along θ=0°, ii) superficial zone fibrils by a “belt”-like Gaussian distribution centred at θ=π/2 and equally distributed around φ and iii) the narrow transitional zone by an isotropic distribution in 3D with *w*(*θ*,*φ*) = 1/4π. **Figure 2A** shows the real- and reciprocal space distributions for these distinct fibril orientation distributions. Experimentally, the centres of the fibril angular distributions were not precisely at 0° (deep zone) and π/2 (superficial) but showed slight variations of a couple of degrees which is expected. To facilitate the nonlinear fit algorithm it was found practical to shift the peak of the distribution to exactly along 0 (deep zone and calcified plate) and π/2 (superficial zone; since transitional zone had no peak this consideration was not relevant there).

The axial width w_a_ can be linked to the natural variance in D at each scattering volume, denoted ΔD, if the effects from beam broadening of the reflections are not significant. Since q_0_ = 6π/D, we have ΔD= D^2^w_a_/(3π) ~ 0.9 nm for w_a_~0.002 nm^-1^ and D~66.8 nm.

**Section S2: Data representation**:

For data binning, the *pandas* “*cut”* function from the Python *pandas* library was used to bin the aggregated data in 5% fractional depth steps. For the confidence intervals in **Figure 7**, ellipsoidal confidence intervals were calculated for the DZ, SZ, TZ regions separately (for AC) and for AC and CP (for the whole sample) using as a basis the code example given on the Matplotlib website (<https://matplotlib.org/stable/gallery/statistics/confidence_ellipse.html>). For the confidence interval representation only, for visual clarity, data for each group or zone was treated to remove outliers by calculating the mean and standard deviation for the x- and y-data and applying a 3z-score to remove points > 3 S.Ds away from the mean in both cases. The covariance confidence ellipse is calculated and plotted, as shown in **Figure 7**. The *mgcv* package in R was used to calculate generalized additive models of the variation of fibrillar parameters with loading and distance from the joint surface (see **Section S4**).

**Section S3: Model of nanoscale kinking and disordering**:

A simplified 1D model for the fibril kinking is described. Consider the fibrils to be straight and vertical (approximately true in the deep zone), and to comprise of a series of crystalline and noncrystalline segments (as in **Figure 6**). In the undeformed state, over a region *L*, and length of the crystalline segments *l*_c_, *L* = *N* *l*_c_ (likewise, NC: noncrystalline). In the deformed state, the length of this domain is shortened to *L*_ε_ = *N*_c_ *l*_c_ + *N*_nc_ *l*_nc_ = *L* – *N*_nc_(*l*_c_-*l*_nc_) < L. The linear density of crystalline domains is:

$\rho_{\varepsilon}=\frac{N_{c}}{Nl_{c}-N_{nc}\left( l_{c}-l_{nc} \right)}$

Assuming the scattering per fibril unit is the same before and after compression for the crystalline segments (i.e. that $\left( \rho_{O}-\rho_{G} \right)^{2}$ does not change), the scattering over a fixed region *b* (denoting beam-size) is proportional to the number of crystalline segments at a strain level ε, i.e. $I_{\varepsilon}\sim kn_{\varepsilon}$. Since $n_{0}=\frac{b}{l_{c}}$, we have

$$I_{\varepsilon}\sim kb\frac{N_{c}}{Nl_{c}-N_{nc}\left( l_{c}-l_{nc} \right)}<k\frac{b}{l_{c}}=kn_{0}=I_{0}$$

i.e. $I_{\varepsilon}<I_{0}$ as expected. The fractional change in intensity is:

$$\frac{\delta I_{0}}{I_{0}}=\frac{-N_{nc}l_{nc}}{Nl_{c}-N_{nc}\left( l_{c}-l_{nc} \right)}$$

Define two parameters 1) the noncrystalline fraction $r_{N}=\frac{N_{nc}}{N}$ and 2) length ratio $r_{l}=\frac{l_{nc}}{l_{c}}$.

The linear density in the compressed case is:

$$\rho_{\varepsilon}=\frac{1}{l_{c}}\frac{1-r_{N}}{1-r_{N}\left( 1-r_{l} \right)}$$

The linear density does not change if *r*_l_ = 1, as expected.

The strain ε can be expressed in terms of these parameters:

$$\varepsilon=\frac{L_{\varepsilon}-L}{L}=\frac{Nl_{c}-N_{nc}\left( l_{c}-l_{nc} \right)-Nl_{c}}{Nl_{c}}=\frac{-N_{nc}\left( l_{c}-l_{nc} \right)}{Nl_{c}}=-r_{N}\left( 1-r_{l} \right)$$

The fractional change in intensity is:

$\frac{\delta I_{0}}{I_{0}}=\frac{-r_{N}r_{l}}{1-r_{N}\left( 1-r_{l} \right)}=\frac{-r_{N}r_{l}}{1+}$ **(S1)**

In this framework, the (<0.5%) changes in D-period (which reflect in a change in *l*_c_) are considered negligible relative to the measured ~20-40% tissue strain.

*Radial compaction linked to kinking*: In our model, the reduction in fibril diameter can be linked to the presence of kinked non-crystalline segments. We take the radius of the noncrystalline segments R_NC_ < R_C_ (radius of crystalline segments), due to loss of intrafibrillar water. The length fractions f_C_, f_NC_ of the crystalline and noncrystalline segments can be calculated from the length of a deformed region $L_{\varepsilon}=N_{C}l_{C}+N_{NC}l_{NC}$ by:

$$f_{C}=\frac{N_{C}l_{C}}{N_{C}l_{C}+N_{NC}l_{NC}};f_{NC}=\frac{N_{NC}l_{NC}}{N_{C}l_{C}+N_{NC}l_{NC}}$$

which can be also expressed in terms of the number fractions defined earlier:

$$f_{C}=\frac{1-r_{N}}{1-r_{N}\left( 1-r_{l} \right)};f_{NC}=\frac{r_{N}r_{l}}{1-r_{N}\left( 1-r_{l} \right)}$$

The average radius measured by SAXS is the length-weighted sum of the two segments:

$$R=R_{C}f_{C}+R_{NC}f_{NC}$$

and the transverse fibril strain is:

$$\varepsilon_{R}=\frac{\Delta R}{R}=\frac{R_{C}f_{C}+R_{NC}f_{NC}-R_{C}}{R_{C}}=f_{C}+\frac{R_{NC}}{R_{C}}f_{NC}-1$$

Since in the model, the kinked (compressed) segments lose intrafibrillar water, the ratio *R*_NC_/*R*_C_ can be taken as κ = 1.1 nm/1.5 nm, based on estimates of lateral tropocollagen molecule spacing in dehydrated and hydrated tissues ^[3]^, leading to:

$f_{C}\left( r_{N},r_{l} \right)=-\kappa f_{NC}\left( r_{N},r_{l} \right)+1+\varepsilon_{R}$ **(S2)**

where the brackets denote that the terms are functions of the ratios defined earlier. In equations *(S1)* and *(S2)*, the fractional change in intensity, radius κ, and strains (ε and ε_R_) are experimentally observable, while r_N_ and r_l_ are model parameters which can be solved for, giving:

$r_{N}=1-\frac{\delta I_{0}}{I_{0}}\frac{1+\varepsilon}{\varepsilon_{R}}\left( 1-\kappa+\varepsilon_{R} \right)$ **(S3)**

and

$r_{l}=-\varepsilon_{R}\frac{\delta I_{0}}{I_{0}}\frac{1+\varepsilon}{\varepsilon_{R}}\frac{1}{r_{N}}$ **(S3)**

These relations can be plotted as a function of the fractional change in intensity δI_0_/I_0_, for different combinations of axial and radial strains, as shown in **Figure S10**.

| **Geometrical model of fibrillar water loss and disordering** | |
| --- | --- |
| $\zeta_{I}=\frac{-r_{N}r_{l}}{1+\varepsilon_{T}}$ (1) | $r_{N}=1-\zeta_{I}\frac{1+\varepsilon_{T}}{\varepsilon_{R}}\left( 1-\kappa+\varepsilon_{R} \right)$ (2) |
| $r_{l}=-\varepsilon_{R}\frac{-\zeta_{I}\frac{1+\varepsilon_{T}}{\varepsilon_{R}}}{1-\zeta_{I}\frac{1+\varepsilon_{T}}{\varepsilon_{R}}\left( 1-\kappa+\varepsilon_{R} \right)}$ (3) | $f_{NC}=\frac{r_{N}r_{l}}{1-r_{N}+r_{N}r_{l}}$ (4) |

**Table S1**: Parameters for the fibril kinking model

**Intensity ratios in terms of disorder and gap/overlap ratio**:

The overlap and gap lengths in a fibril (**Figure 6B**) are denoted O and G, respectively, with O + G = D. Using a one-dimensional model of the electron density profile as a step-function (high in the overlap and low in the gap zones), the peak intensity ratio of the *n*^th^ to the *m*^th^ peak area can be written as (*n*, *m* are integers denoting the peak order):

$$\frac{I_{m}}{I_{n}}=\left( \frac{n}{m} \right)^{2}\left( \frac{\sin\left( m\pi\left( O/D \right) \right)}{\sin\left( n\pi\left( O/D \right) \right)} \right)^{2}$$

To characterise the blurring of the sharp gap/overlap interface by random intrafibrillar axial shifts in tropocollagen molecules, the peak intensities are reduced by a Debye-Waller type term exp(- κ q^2^) as described earlier ^[4]^, leading to:

$$\frac{I_{m}}{I_{n}}=\left( \frac{n}{m} \right)^{2}\left( \frac{\sin\left( m\pi\left( O/D \right) \right)}{\sin\left( n\pi\left( O/D \right) \right)} \right)^{2}\exp\left( -\kappa\left( \frac{2\pi}{D} \right)^{2}\left( m^{2}-n^{2} \right) \right)$$

Using a reference peak intensity (n=3), the intensity ratios can be calculated for a series of visible peaks n=3,5,6,7,8,9,10,11. Examples of the SAXS I(q) profiles before and after loading are shown in **Figure S9A**. The I(q) profiles were binned in steps of 5% fractional depth, averaged, and the area under the different peak orders calculated for *I*_n_ (n=3,5,6,7,8,9,10,11).

**Section S4: Generalized additive model analysis**

To check for spatial trends in the SAXS parameters, and changes induced by loading, data were fitted to generalized additive models (GAMs) using the *mgcv* package in R (Wood S.N. (2017) *Generalized Additive Models: An Introduction with R (2nd edition)*. Chapman and Hall/CRC Press). The general formula “*[parameter] ~ s(scaled distance, by = loading) + loading*” and spline curves were used for the smooth components. Parameters include D, fibril diameter, w_a_, w_χ_, I_0_, and rho. All GAM models were checked after fitting using the gam.check() function to check model convergence, sufficient basis functions and normality of residual distribution. A summary of the key parameters (parametric coefficients and their significance, and significance of smooth terms) is provided for each parameter below. The curves are not shown in the main **Figure 4** to avoid overcrowding the figures.

**Section S5: Radiation Damage**

To check that radiation damage was not causing the changes observed (e.g. intensity reductions on compression), 1) we calculated the radiation dose D and 2) carried out repeated 1s exposures on the articular cartilage to see the onset of changes in the SAXS pattern, reflecting radiation-induced ultrastructural changes.

For 1), a single point was repeatedly measured with the X-ray beam with 1s exposure times consecutively, to track when reductions of peak intensity and other changes in peak characteristics occurred. This is distinct from, and a more severe treatment relative to, the scanning protocol, and is hence used to establish an upper limit for the potential damage. Each scan location is measured twice but only after the intervening points have been scanned, i.e. with a 20–30-minute gap between measurements, and this waiting time has been found empirically to reduce rate of damage buildup. From **Figure S7A**, peak intensity starts decreasing by the 3^rd^ consecutive 1s exposure at the same point.

In our protocol, the sample is scanned 2×. However, since a sparser grid step-size (40 μm) than the beam size (20 μm) was used, the per-location tissue exposure is <2×, which is below the level at which changes are visible in the SAXS pattern (**Figure S8A**, left shaded region). We simulated tissue displacement versus measurement grids of spacings of 40 μm and beam-size of 20 μm, to calculate how much each tissue zone is exposed to the beam after unloaded and loaded-scans (**Figure S7B**). An average of 1.5× of tissue exposure was observed, below the level (3x and higher) at which peak intensity reductions are observed in the consecutive exposure test. The exposure level remains around 1.5 when small displacements at the calcified cartilage/articular cartilage interface are considered as well.

For 2), the radiation dose D was estimated as described previously on related in situ SAXS on collagenous tissues ^[5]^

$$D=\frac{I_{0}.\tau.A.E}{\Delta_{x}\Delta_{y}\Delta_{z}.\rho}$$

where the incident flux I_0_, A is the absorption ratio, E the beam energy, τ the exposure time, Δ_x_Δ_y_Δ_z_ is the scattering volume (beam area x sample thickness) and ρ the density of the sample estimated as 1100 kg.m^-3^).

From the above, the radiation dose/point per scan is 12 kGy. While the scan is done twice on the sample, due to tissue displacement by compression combined with scan spacing of 40 μm, there is an offset and the same tissue points are not fully exposed twice. Simulations of the displacement show the scanned tissue locations are exposed between 1.0x and 2.0x times, with an average of 1.5x (**Figure S7B**). Total radiation exposure/point is thus between 12-24 kGy with a total average of 18 kGy over two 1s exposures, with a wait period of 60 min between the 1s exposures (time between consecutive SAXS scans). This dose level is consistent with our repeat SAXS exposures on the tissue (**Figure S7A**), showing the onset of damage (5% drop in meridional peak intensity) at 3.0x exposures (with no wait period) and increasing thereafter because, by 3.0x exposures, the tissue point would have been exposed to ~36 kGy, close to the threshold of 35 kGy seen for damage in bone ^[6]^. Hence we do not expect radiation damage to the tissue to significantly contribute to our results. Our results for the used exposure rate are consistent with e.g. prior studies of in situ loading and SAXS on other soft tissues like tendon ^[5]^, where rates of 6-7 kGy/s showed minimal changes in collagen ultrastructural properties, but higher doses of 106-124 kGy/s showed reductions in peak area by 1s. In earlier studies on collagen from breast cancer tissue ^[7]^, a similar dose rate (10 kGy/s) was used, and changes were seen only after 100 kGy total dose (i.e. after 10 s), which is well beyond the 2×1s exposures used in the current work.

**Section S6: Tilting versus I_0_ changes**:

The 3D model in S1 can be calculated when the entire fibril distribution is tilted out of plane, e.g. as in the case shown in **Figure 2C1** bottom. The measured SAXS pattern will be the intersection of the reciprocal space intensity with the Ewald sphere (plane in SAXS case). By computing these 2D patterns and integrating azimuthally and radially, the equivalent I(q) and I(χ) plots can also be obtained.

Here, we tested how a combination of out of plane tilting, and increase in angular width w_χ_, would affect the peak intensities. The calculation is to test the alternative scenario where the measured drops in SAXS peak intensities (**Figure S9A**) come about due to this tilting effect, rather than a change in the number of crystalline fibrils. The tilting refers here to the average fibril direction, within the microscale scattering volume, tilting out of the 2D plane normal to the X-ray beam. If the number of crystalline fibrils reduces, but the orientation stays the same, there will be no change in angular peak position and the intensities will go down on average (in a vertical direction at the abscissa zero point in **Figure S9B**).

If there is out of plane tilt, however, the measured 2D angular peak position from I(χ) may change, although it does not give the 3D orientation. There will be a combination of an angular shift and decrease in relative peak intensity, i.e. the experimental data lying on a right/downward sloping curve whose gradient depends on the total angular change occurring. Since any changed 3D orientation is not straightforward to determine using 2D SAXS, an indirect approach was used, with the angular position of the I(χ) peaks was used as a projected angle to estimate the angular tilt. The results are shown in **Figure S9B**. To match experiment, w_χ_ varied from 0.20 radians (undeformed) to 0.26 radians (deformed), corresponding to the ~30% increase observed in the deep zone between 60-80% (**Figure 5**). The data lies somewhere in between the pure downward shift and the model trajectories for angular shift alone, indicating that the real effect may be a combination of both factors.

**Supplementary Figures**:


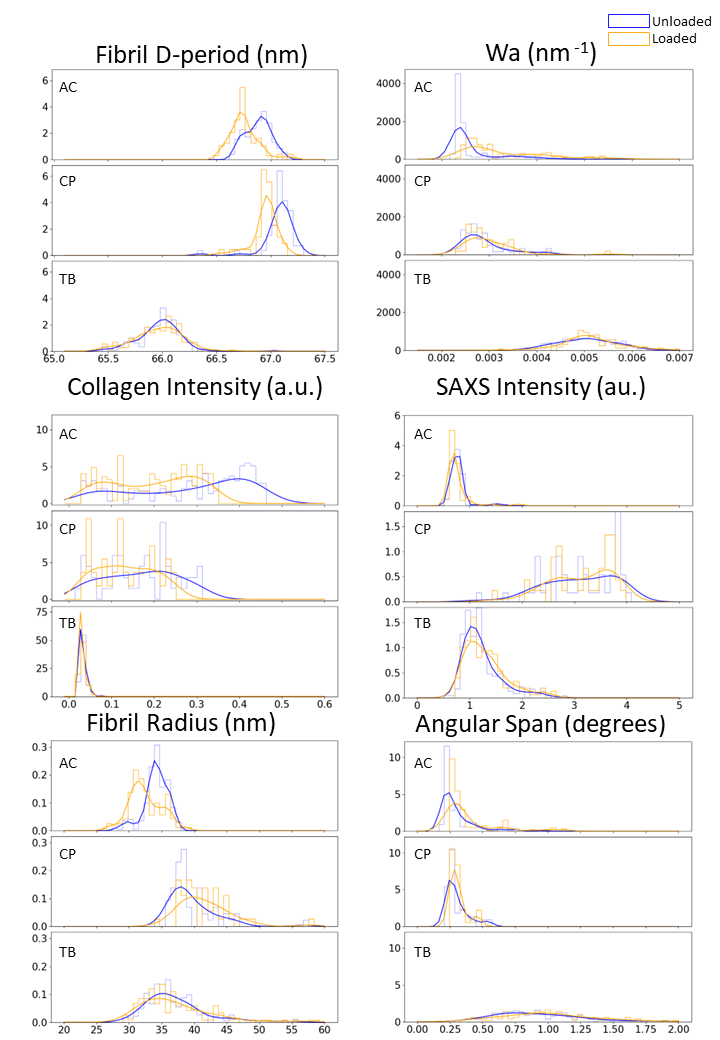


**Figure S1**: Pre (blue) and post (orange) histograms and kernel density estimators (KDEs) demonstrating the shift in fibril nanomechanical parameters upon loading, divided according to zone (AC: articular cartilage, CP: calcified plate and TB: trabecular bone).


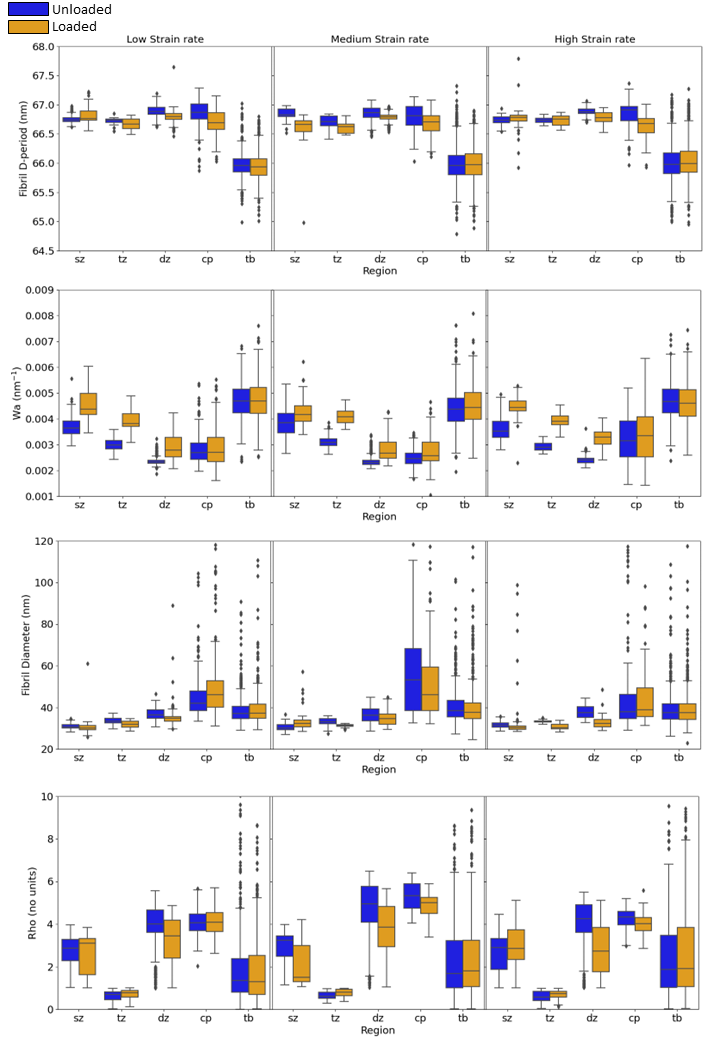


**Figure S2**: Comparison of pre-(blue) and post-loading (orange) fibril parameters as a function of initial loading rate (to reach target strain). No clear difference is observable between loading rates.


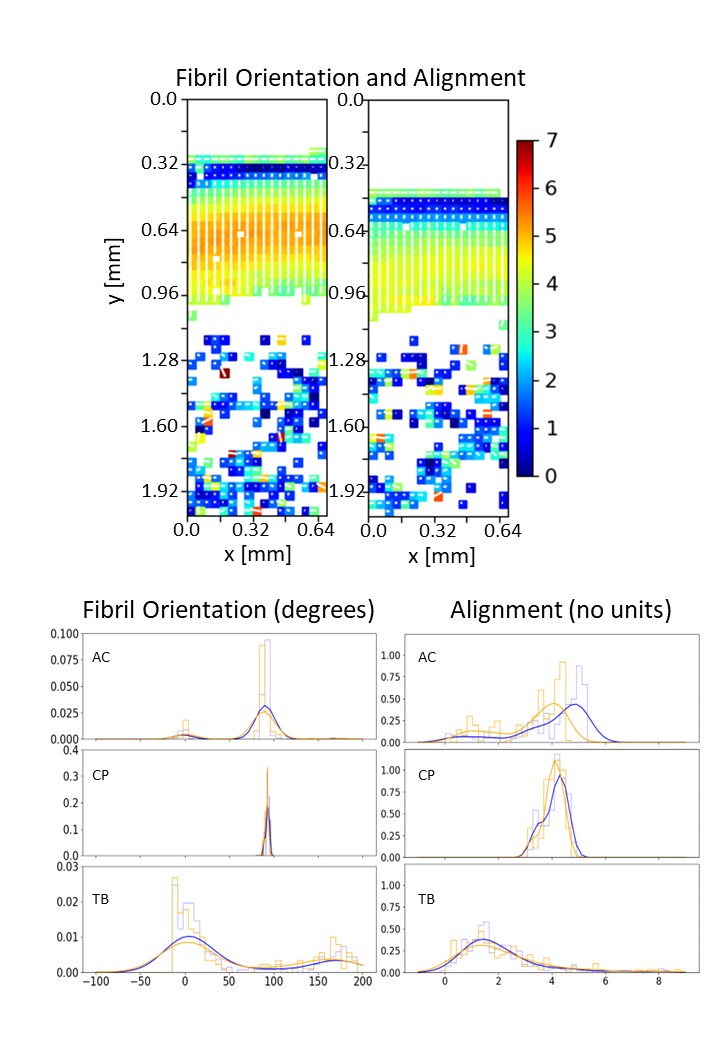


**Figure S3**: Colormap of the direction (white lines) and degree of alignment (inversely related to w_χ_), complementing Figure 3. Histograms and KDEs below on a zone-specific basis. Load-induced changes are observed mainly in the articular cartilage, and mainly in the degree of alignment rather than the direction.


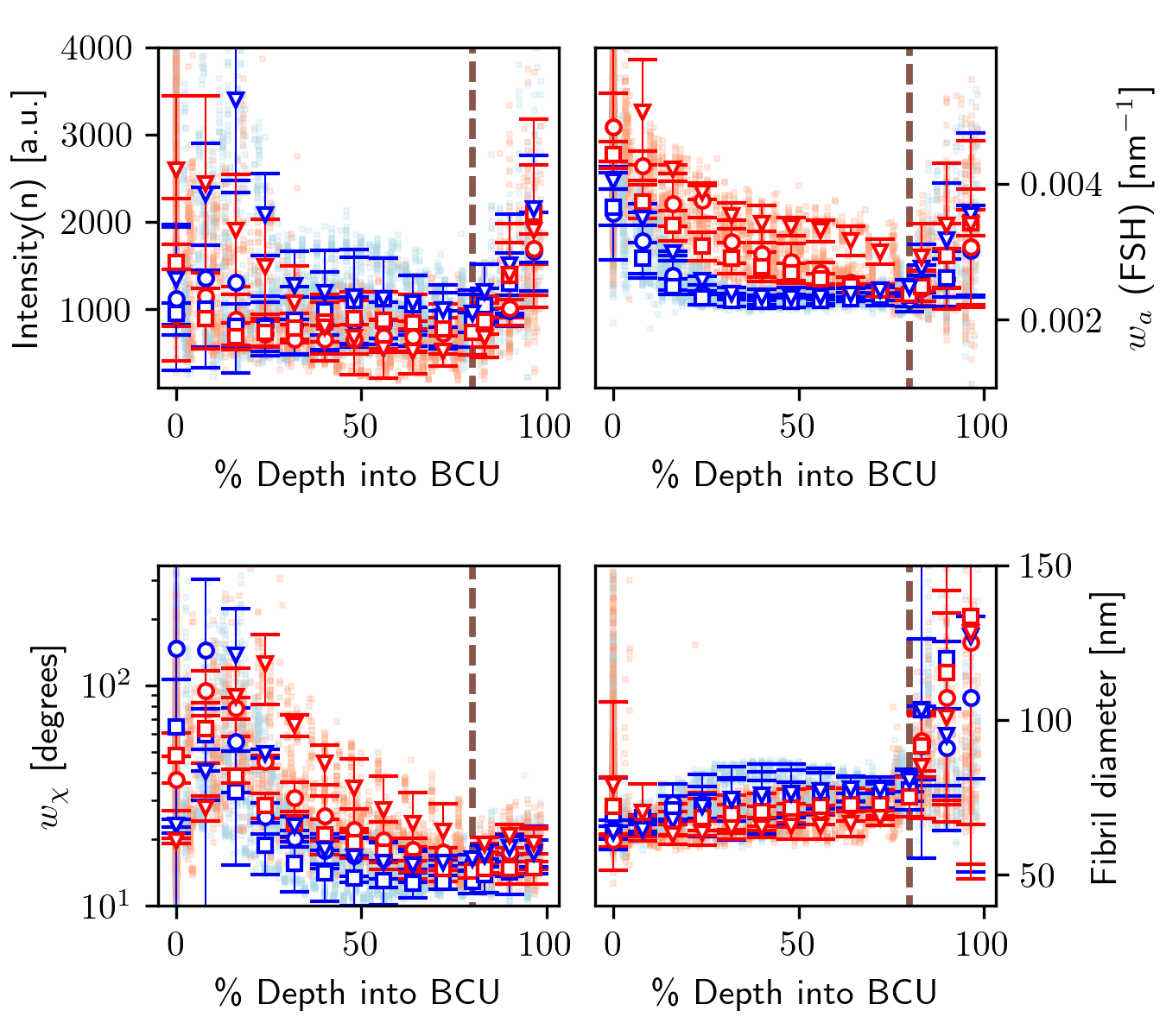


**Figure S4**: Changes in scattering intensity pre-factor I_0_ before (blue) and after (red) loading, using a similar labelling convention as in **Figure 4**, but with all strain-rates merged into a single plot (different symbols correspond to different strain rates: circles: low; squares: medium; triangles: high). Reduction in I_0_ is observed with greater effects for the high strain-rate group (which was deformed to a larger level), as expected.


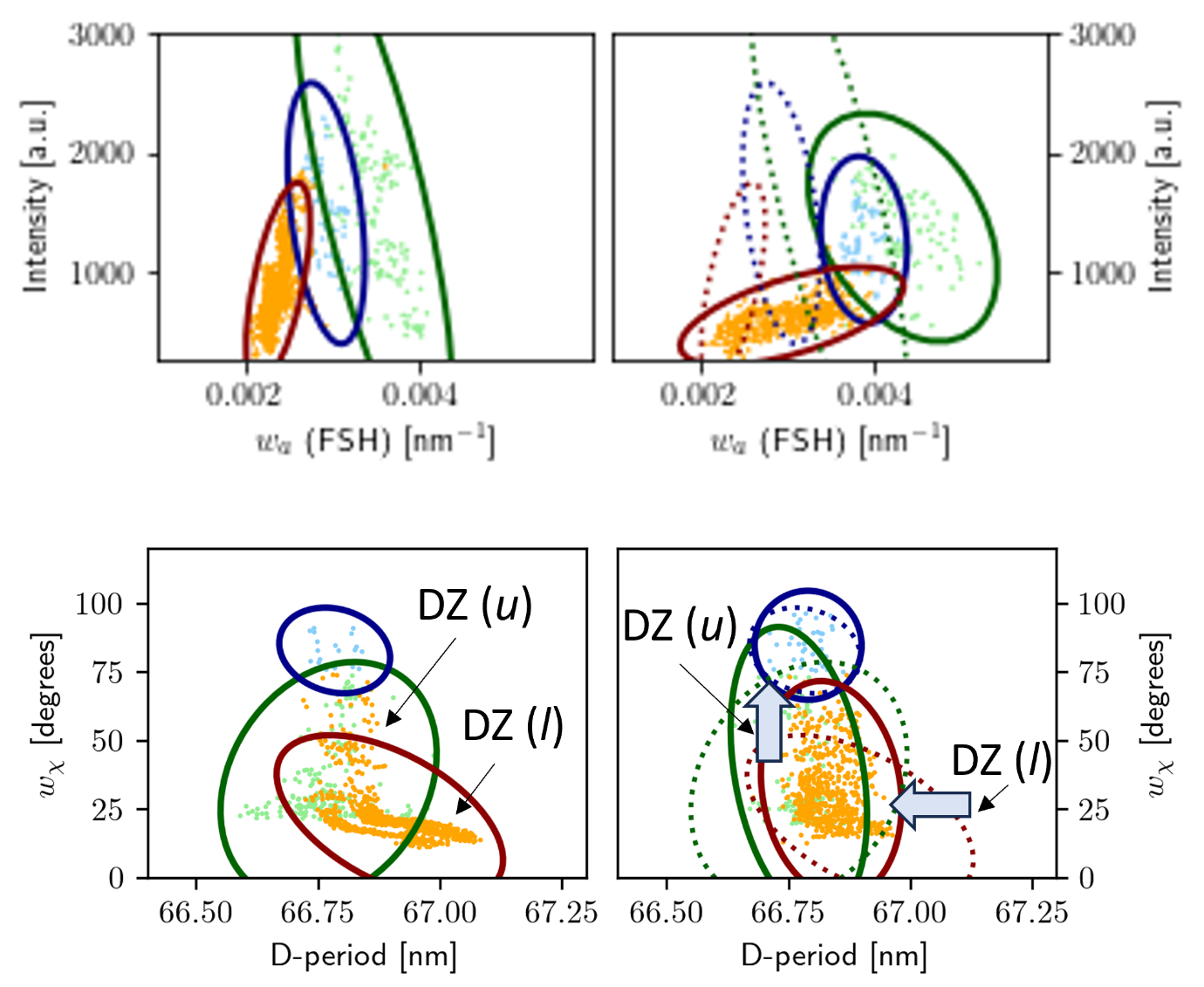


**Figure S5**: Correlation plot for I_0_-w_a_ and w_χ_-w_a_, analogous to **Figure 7**. Changes in I0 are most prominent in deep zone (top). A spatial separation of the response is seen for w_χ_-w_a_ with regions in the DZ closer to the joint surface becoming increasingly misaligned (up arrow) whilst regions toward the calcified plate increase in strain heterogeneity (w_a_).


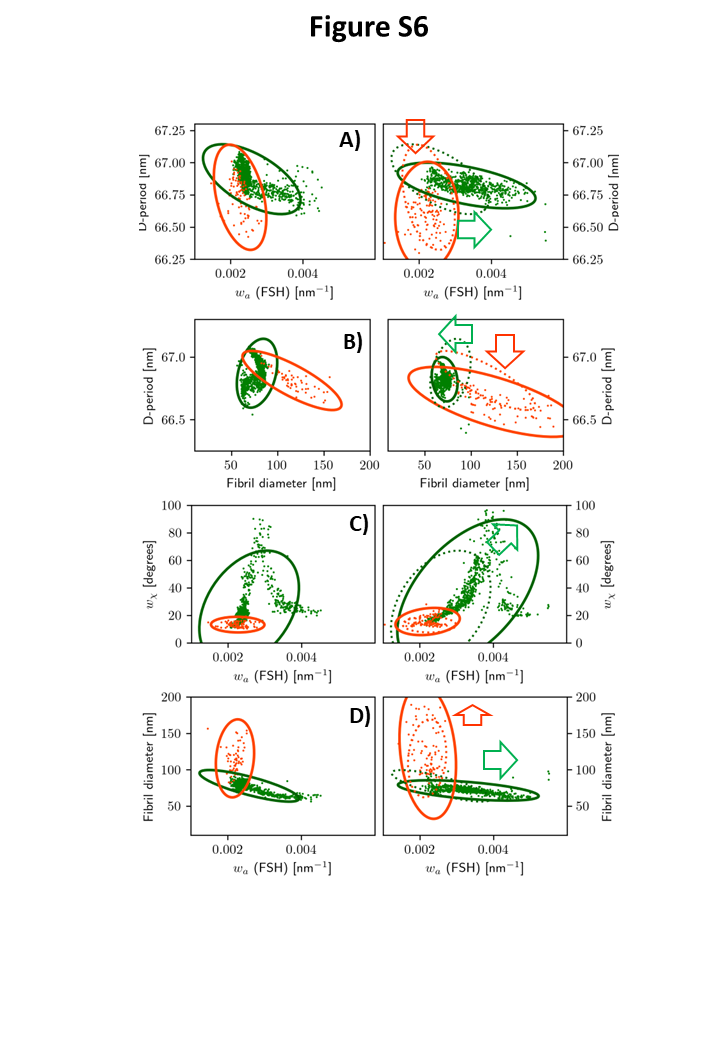


**Figure S6**: Cluster analysis (analogous to **Figure 7**) comparing the articular cartilage (green) with the calcified plate (orange). The opposite trends in fibril diameter versus similar trend in D-period are noted. Minimal changes in strain heterogeneity are observed in the calcified plate.


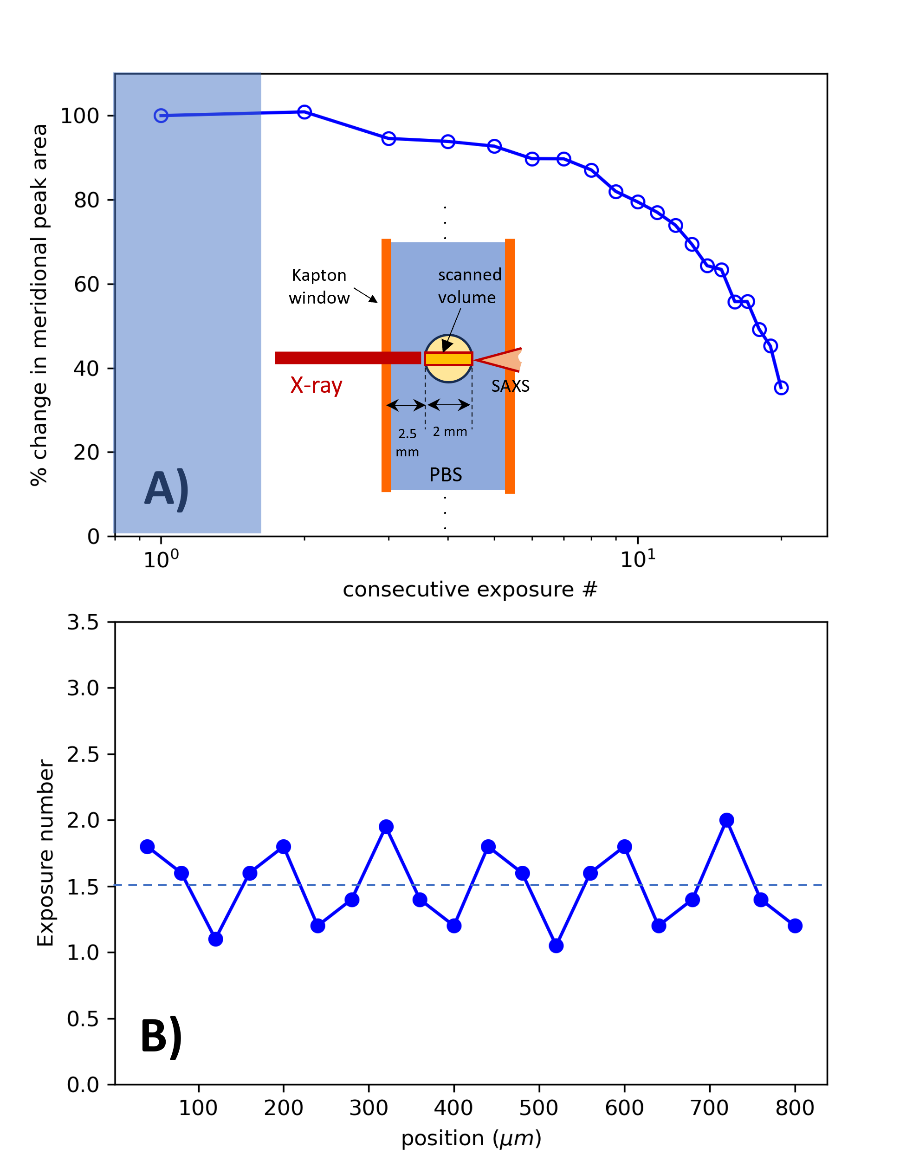


**Figure S7**: *Radiation damage test*: top: A) relative peak intensity change after consecutive exposures to same location. Shaded region – fraction of tissue exposure in the unstressed/loaded scan protocol used. Inset: fluid chamber with cylindrical BCU viewed from above, showing beam path. The scan area is shown as a red rectangle from top – each SAXS pattern is from cylinders through this volume. B) fraction of tissue exposure after pre-load and loaded scans, with the scan parameters used in the experiment.


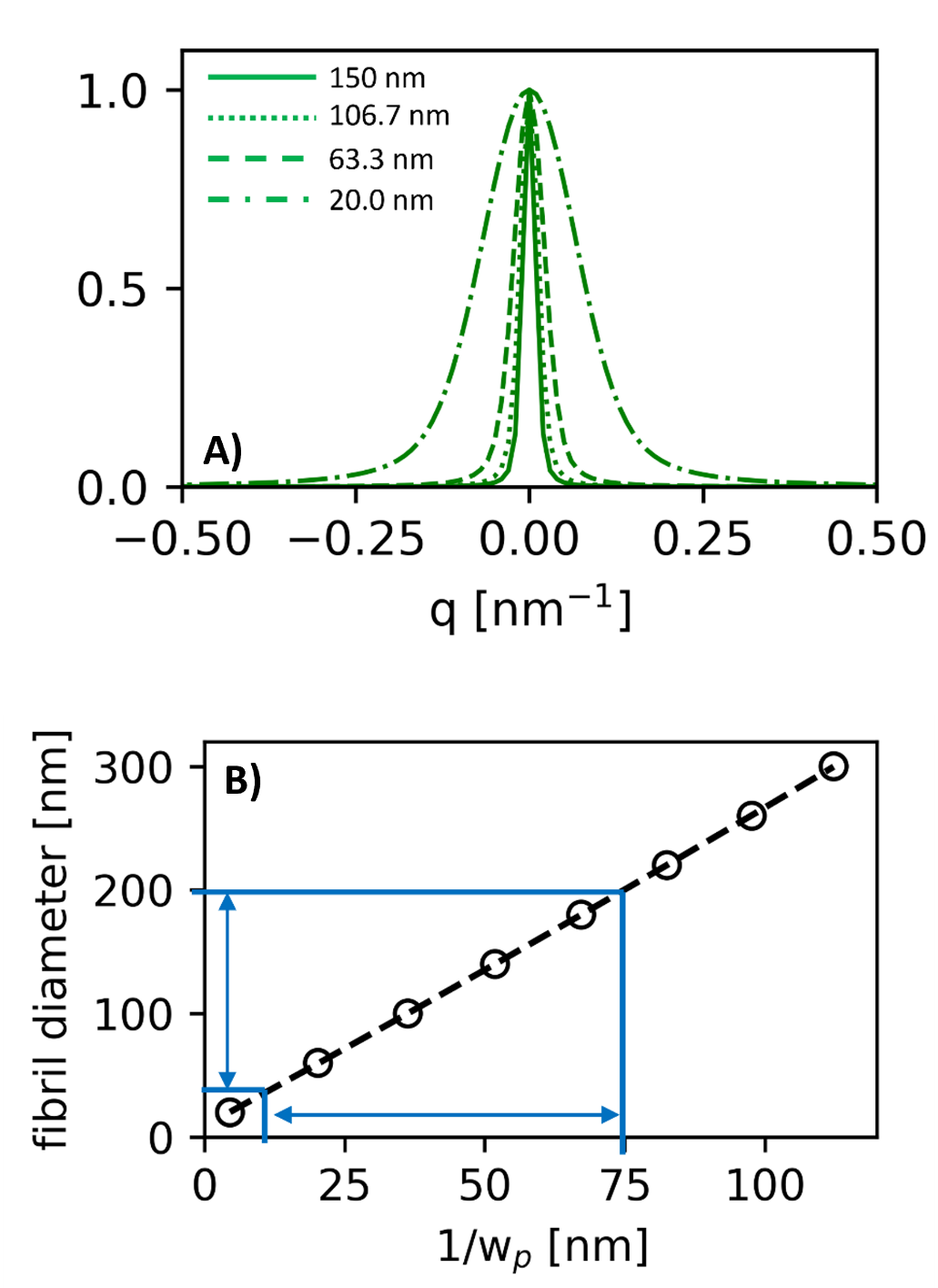


**Figure S8**: *Model calibration*: A) Model SAXS intensity profiles transverse to the fibril axis for average radii from 20.0 nm to 150 nm, showing the narrowing of the equatorial width with increasing radius. B) Second-order polynomial fit between mean fibril diameter and 1/w_p_. The blue lines indicate the expected experimental range of fibril diameters in type II and I cartilage. The near-linear relationship is noted but due to slight nonlinearity at larger 1/w_p_ a second order rather than first-order polynomial is used


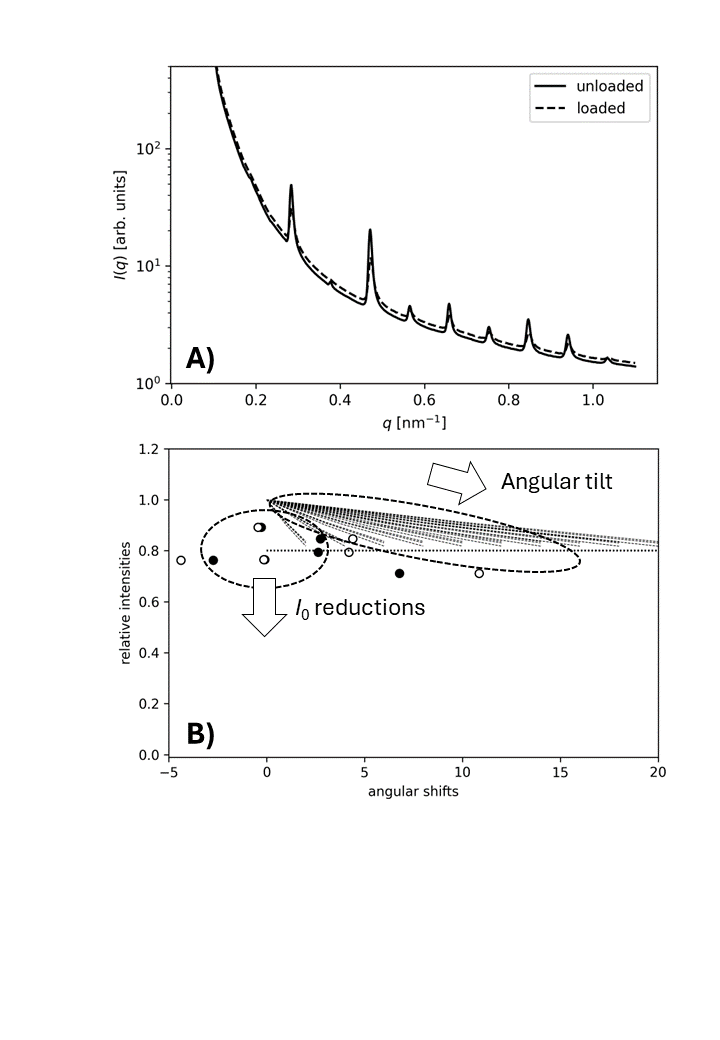


**Figure S9**: *I_0_ reduction on compression and comparison to out of plane tilting simulations*: A) Example *I*(*q*) plots in unloaded (solid) and loaded (dashed) conditions, averaged across a part of the deep zone (60-70%) for one sample. The reduction in meridional peak intensity is evident. B) Simulated changes in relative meridional peak intensity change arising from out-of-plane angular tilting, using the 3D model, with tilts ranging from 2° to 20° (left to right). Each group of dashed lines corresponds to relative intensity changes for orders n=3, 5 and 9; minimal differences are observed between orders. Symbols denote experimental relative intensity changes (low- and medium-strain rates only, as the high strain-rate samples were deformed to higher strain levels). Filled: experimental angular peak shift from 2D I(χ) plots; open: extrapolated 3D solid angle shift. Dashed ellipses: predicted shifts in intensities for i) I_0_ changes with no angular tilting of the fibril distribution and ii) intensity reduction solely due to out-of-plane tilting and the (experimentally observed) increase in angular distribution parameter w_χ_.


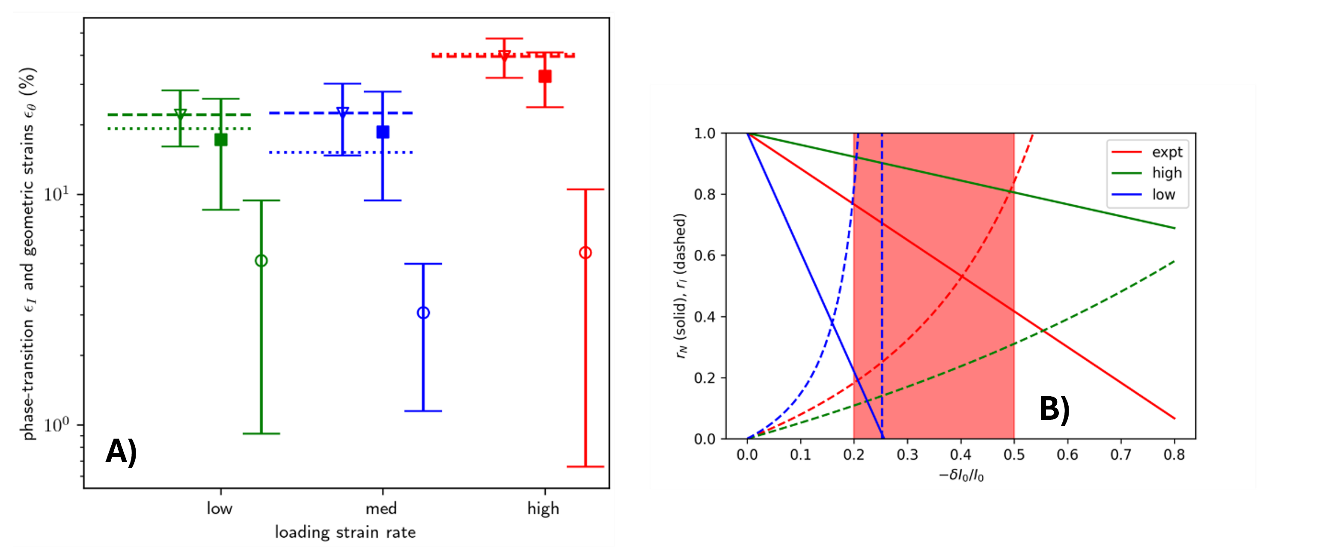


**Figure S10**: *Crystalline-noncrystalline fibrillar transitions contributions to tissue strain:* A) Horizontal dashed lines, open diamonds and error-bars: tissue-level strain calculated from X-ray microfocus maps at each strain rate; filled squares: average % change in I_0_; open circles: averaged geometric strain ε_G_. Dotted lines: predictions for % I_0_ using the compression-induced crystalline-noncrystalline phase transition model of collagen fibrils, after solving for r_N_ and r_L_ using measured tissue strains, intensity changes and radial contractions; good agreement is seen. B) Variation in number of non-crystalline segments r_N_ and length ratios between non-crystalline and crystalline segments r_l_ with increasing magnitude of relative intensity (δI_0_/I_0_) change (reduction). Shaded area shows region of experimentally observed intensity reduction; red lines are typical experimental values for ε and ε_R_ from **Figure 5**.

**Supplementary Data: GAM fits**:

Parameters of the generalized additive models obtained as described in Section S4.

| *rate* | *sample* | *parameter* | *Intercept* |  |  | *loadungUL* | |  |  | *smoothL* |
| --- | --- | --- | --- | --- | --- | --- | --- | --- | --- | --- |
|  | **544963** |  | *av.* | *s.d.* | *p* | *av.* | *s.d.* | *p* | *p* | *p* |
| low | 544963 | D | 66.79 | 0.00 | 2e-16 *** | 1.28E-01 | 6.08E-03 | 2e-16 *** | 2e-16 *** | 2e-16 *** |
| low | 544963 | wa | 3.26E-03 | 1.43E-05 | 2e-16 *** | -5.92E-04 | 1.93E-05 | 2e-16 *** | 2e-16 *** | 2e-16 *** |
| low | 544963 | wd | 32.95 | 6.527 | 6.18E-07 | 6.95E+00 | 8.84E+00 | 0.432 | 0.0134 | 2e-16 *** |
| low | 544963 | Dia. | 69.88 | 0.33 | 2e-16 *** | 2.39E+00 | 4.48E-01 | 1.51E-07 | 2e-16 *** | 2e-16 *** |
| low | 544963 | rho | 3.36 | 0.02 | 2e-16 *** | 6.13E-01 | 3.28E-02 | 2e-16 *** | 2e-16 *** | 2e-16 *** |
| low | 544963 | I0 | 655.93 | 7.20 | 2e-16 *** | 1.42E+02 | 9.75E+00 | 2e-16 *** | 2e-16 *** | 2e-16 *** |
|  | **544976** |  |  |  |  |  |  |  |  |  |
| low | 544976 | D | 66.73 | 0.01 | 2e-16 *** | 6.73E-02 | 1.45E-02 | 4.52E-06 | 2e-16 *** | 2e-16 *** |
| low | 544976 | wa | 3.07E-03 | 4.92E-05 | 2e-16 *** | -4.85E-04 | 6.77E-05 | 2.22E-12 | 2e-16 *** | 2e-16 *** |
| low | 544976 | wd | 33.525 | 7.863 | 2.32E-05 | 9.80E+00 | 1.08E+01 | 0.366 | 0.0253 | 2e-16 *** |
| low | 544976 | Dia. | 79.66 | 0.92 | 2e-16 *** | 3.38E-01 | 1.26E+00 | 0.789 | 2e-16 *** | 2e-16 *** |
| low | 544976 | rho | 3.35 | 0.02 | 2e-16 *** | 3.04E-01 | 3.32E-02 | 2e-16 *** | 2e-16 *** | 2e-16 *** |
| low | 544976 | I0 | 395.92 | 6.61 | 2e-16 *** | -1.96E+01 | 9.11E+00 | 0.0322 | 2e-16 *** | 2e-16 *** |
|  | **545020** |  |  |  |  |  |  |  |  |  |
| low | 545020 | D | 66.81 | 0.00 | 2e-16 *** | 6.96E-02 | 3.17E-03 | 2e-16 *** | 2e-16 *** | 2e-16 *** |
| low | 545020 | wa | 3.14E-03 | 1.01E-05 | 2e-16 *** | -6.08E-04 | 1.35E-05 | 2e-16 *** | 2e-16 *** | 2e-16 *** |
| low | 545020 | wd | 35.52 | 0.659 | 2e-16 *** | -6.13E+00 | 8.75E-01 | 5.53E-12 | 2e-16 *** | 2e-16 *** |
| low | 545020 | Dia. | 76.21 | 0.80 | 2e-16 *** | 2.99E+00 | 1.06E+00 | 0.00475 | 2e-16 *** | 2e-16 *** |
| low | 545020 | rho | 2.83 | 0.01 | 2e-16 *** | 6.69E-01 | 1.92E-02 | 2e-16 *** | 2e-16 *** | 2e-16 *** |
| low | 545020 | I0 | 663.54 | 43.85 | 2e-16 *** | 2.77E+02 | 5.83E+01 | 2.40E-06 | 1.00E-06 | 2e-16 *** |
|  | **544968** |  |  |  |  |  |  |  |  |  |
| med | 544968 | D | 66.75 | 0.01 | 2e-16 *** | 5.95E-02 | 1.38E-02 | 1.93E-05 | 8.27E-05 | 2e-16 *** |
| med | 544968 | wa | 2.92E-03 | 3.23E-05 | 2e-16 *** | -4.27E-04 | 4.56E-05 | 2e-16 *** | 2e-16 *** | 2e-16 *** |
| med | 544968 | wd | 25.3126 | 0.9811 | 2e-16 *** | -2.23E+00 | 1.43E+00 | 0.119 | 2e-16 *** | 2e-16 *** |
| med | 544968 | Dia. | 70.04 | 0.91 | 2e-16 *** | 3.68E+00 | 1.31E+00 | 0.00515 | 2e-16 *** | 2e-16 *** |
| med | 544968 | rho | 3.72 | 0.03 | 2e-16 *** | 1.04E+00 | 3.71E-02 | 2e-16 *** | 2e-16 *** | 2e-16 *** |
| med | 544968 | I0 | 561.69 | 32.34 | 2e-16 *** | -5.84E+01 | 4.49E+01 | 0.194 | 2e-16 *** | 0.00078 |
|  | **544980** |  |  |  |  |  |  |  |  |  |
| med | 544980 | D | 66.75 | 0.00 | 2e-16 *** | 1.13E-01 | 5.62E-03 | 2e-16 *** | 2e-16 *** | 2e-16 *** |
| med | 544980 | wa | 2.97E-03 | 1.49E-05 | 2e-16 *** | -5.44E-04 | 2.06E-05 | 2e-16 *** | 2e-16 *** | 2e-16 *** |
| med | 544980 | wd | 25.1759 | 0.7171 | 2e-16 *** | -6.37E+00 | 9.94E-01 | 2.97E-10 | 2e-16 *** | 2e-16 *** |
| med | 544980 | Dia. | 71.91 | 0.83 | 2e-16 *** | 3.71E+00 | 1.15E+00 | 0.00131 | 2e-16 *** | 0.0468 |
| med | 544980 | rho | 4.03 | 0.03 | 2e-16 *** | 7.41E-01 | 4.50E-02 | 2e-16 *** | 2e-16 *** | 2e-16 *** |
| med | 544980 | I0 | 643.32 | 18.82 | 2e-16 *** | 1.70E+02 | 2.61E+01 | 1.64E-10 | 2e-16 *** | 2e-16 *** |
|  | **545023** |  |  |  |  |  |  |  |  |  |
| med | 545023 | D | 66.80 | 0.02 | 2e-16 *** | 6.17E-02 | 2.72E-02 | 0.0238 | 0.0764 | 0.0194 |
| med | 545023 | wa | 3.04E-03 | 5.24E-05 | 2e-16 *** | -4.75E-04 | 6.95E-05 | 2.00E-11 | 2e-16 *** | 2e-16 *** |
| med | 545023 | wd | 27.5841 | 0.5667 | 2e-16 *** | -3.59E+00 | 7.52E-01 | 2.17E-06 | 2e-16 *** | 2e-16 *** |
| med | 545023 | Dia. | 87.18 | 0.92 | 2e-16 *** | 1.77E+00 | 1.21E+00 | 0.145 | 2e-16 *** | 2e-16 *** |
| med | 545023 | rho | 3.37 | 0.02 | 2e-16 *** | 7.18E-01 | 2.69E-02 | 2e-16 *** | 2e-16 *** | 2e-16 *** |
| med | 545023 | I0 | 721.84 | 12.09 | 2e-16 *** | 6.92E+01 | 1.60E+01 | 1.86E-05 | 2e-16 *** | 2e-16 *** |
|  | **544973** |  |  |  |  |  |  |  |  |  |
| high | 544973 | D | 66.68 | 0.01 | 2e-16 *** | 1.36E-01 | 1.16E-02 | 2e-16 *** | 2e-16 *** | 2e-16 *** |
| high | 544973 | wa | 3.69E-03 | 3.45E-05 | 2e-16 *** | -9.86E-04 | 4.49E-05 | 2e-16 *** | 2e-16 *** | 2e-16 *** |
| high | 544973 | wd | 40.486 | 1.135 | 2e-16 *** | -9.55E+00 | 1.48E+00 | 2.07E-10 | 2e-16 *** | 2e-16 *** |
| high | 544973 | Dia. | 67.46 | 0.51 | 2e-16 *** | 5.69E+00 | 6.65E-01 | 2e-16 *** | 2e-16 *** | 2e-16 *** |
| high | 544973 | rho | 2.79 | 0.02 | 2e-16 *** | 8.99E-01 | 3.14E-02 | 2e-16 *** | 2e-16 *** | 2e-16 *** |
| high | 544973 | I0 | 654.67 | 20.82 | 2e-16 *** | 4.19E+02 | 2.71E+01 | 2e-16 *** | 2e-16 *** | 2e-16 *** |
|  | **544983** |  |  |  |  |  |  |  |  |  |
| high | 544983 | D | 66.82 | 0.02 | 2e-16 *** | 5.18E-02 | 2.19E-02 | 0.0184 | 2e-16 *** | 6.58E-06 |
| high | 544983 | wa | 3.78E-03 | 5.40E-05 | 2e-16 *** | -9.20E-04 | 6.80E-05 | 2e-16 *** | 2e-16 *** | 2e-16 *** |
| high | 544983 | wd | 47.007 | 5.888 | 7.06E-15 | -4.30E+00 | 7.43E+00 | 0.563 | 2e-16 *** | 2e-16 *** |
| high | 544983 | Dia. | 67.31 | 0.50 | 2e-16 *** | 4.03E+00 | 6.31E-01 | 3.49E-10 | 2e-16 *** | 2e-16 *** |
| high | 544983 | rho | 3.08 | 0.03 | 2e-16 *** | 7.61E-01 | 3.36E-02 | 2e-16 *** | 2e-16 *** | 2e-16 *** |
| high | 544983 | I0 | 741.67 | 34.97 | 2e-16 *** | 2.26E+02 | 4.42E+01 | 3.93E-07 | 2e-16 *** | 2e-16 *** |
|  | **545026** |  |  |  |  |  |  |  |  |  |
| high | 545026 | D | 66.82 | 0.01 | 2e-16 *** | 2.47E-02 | 6.34E-03 | 0.000106 | 2e-16 *** | 2e-16 *** |
| high | 545026 | wa | 3.57E-03 | 1.92E-05 | 2e-16 *** | -9.00E-04 | 2.42E-05 | 2e-16 *** | 2e-16 *** | 2e-16 *** |
| high | 545026 | wd | 42.402 | 0.4164 | 2e-16 *** | -1.57E+01 | 5.25E-01 | 2e-16 *** | 2e-16 *** | 2e-16 *** |
| high | 545026 | Dia. | 75.94 | 0.99 | 2e-16 *** | 9.80E+00 | 1.25E+00 | 1.46E-14 | 2e-16 *** | 2e-16 *** |
| high | 545026 | rho | 2.28 | 0.02 | 2e-16 *** | 1.11E+00 | 1.98E-02 | 2e-16 *** | 2e-16 *** | 2e-16 *** |
| high | 545026 | I0 | 985.86 | 26.32 | 2e-16 *** | 3.46E+02 | 3.32E+01 | 2e-16 *** | 2e-16 *** | 2e-16 *** |

**Table S2**: Summary of GAM model results for the D-period (D), axial strain heterogeneity (wa), angular width of distribution (wchi), fibril diameter (Dia.), and prefactor I_0_ (I0).

|  | **ANOVA** | | | **Tukey HSD (Factor)** | | |
| --- | --- | --- | --- | --- | --- | --- |
| **Parameter** | ***p(Factor)*** | ***p(x)*** | ***p***  ***(Factor:x)*** | **Low/Med** | **Low/High** | **Med/High** |
| D | 0.002** | <0.001*** | 0.681 | 0.990 | 0.005** | 0.008** |
| w_a_ | 0.245 | <0.001*** | 0.355 | 0.277 | 0.359 | 0.985 |
| w_χ_ | 0.999 | 0.172 | 0.847 | 0.999 | 0.999 | 0.999 |
| Fibril diameter | 0.857 | 0.605 | 0.988 | 0.964 | 0.952 | 0.844 |

**Table S3**: Summary of 2-way ANOVA tests, and associated Tukey Honestly Significant Difference (HSD) test results for the % change in SAXS ultrastructural parameters in Figure 4. The model is Parameter = Factor * x; Factor is the strain rate (“low”, “med”(medium), “high”), and x is the fractional depth into the BCU (restricted to the articular cartilage). Only the D-period shows a significant difference (p<0.01) with the strain-rate; Tukey post-hoc tests show the difference is between the highest strain-rate and the low and medium strain-rates. *n*=3 samples are in each strain-rate group and the same binned x-range is used for each sample. . We believe that the higher strain achieved experimentally (in error) in the high strain-rate case is the source of this difference in D-period.

| **Strain rate** | **Sample id** | **Tissue strain (%)** |
| --- | --- | --- |
| Low | 544963 | 17.4 |
|  | 544976 | 19.9 |
|  | 545020 | 28.9 |
| Medium | 544968 | 23.9 |
|  | 544980 | 14.1 |
|  | 545023 | 29.3 |
| high | 544973 | 30.7 |
|  | 544983 | 43.3 |
|  | 545026 | 44.4 |

**Table S4**: Average tissue strain in the articular cartilage, measured using the SAXS intensity maps before and after compression.

**Supplementary Mechanical Data**:

The maximum tangent modulus, maximum stress, and relaxation modulus of the tested samples are shown below (**Figure S11**). As expected, there is an increased trend particularly pronounced for the high strain rate.


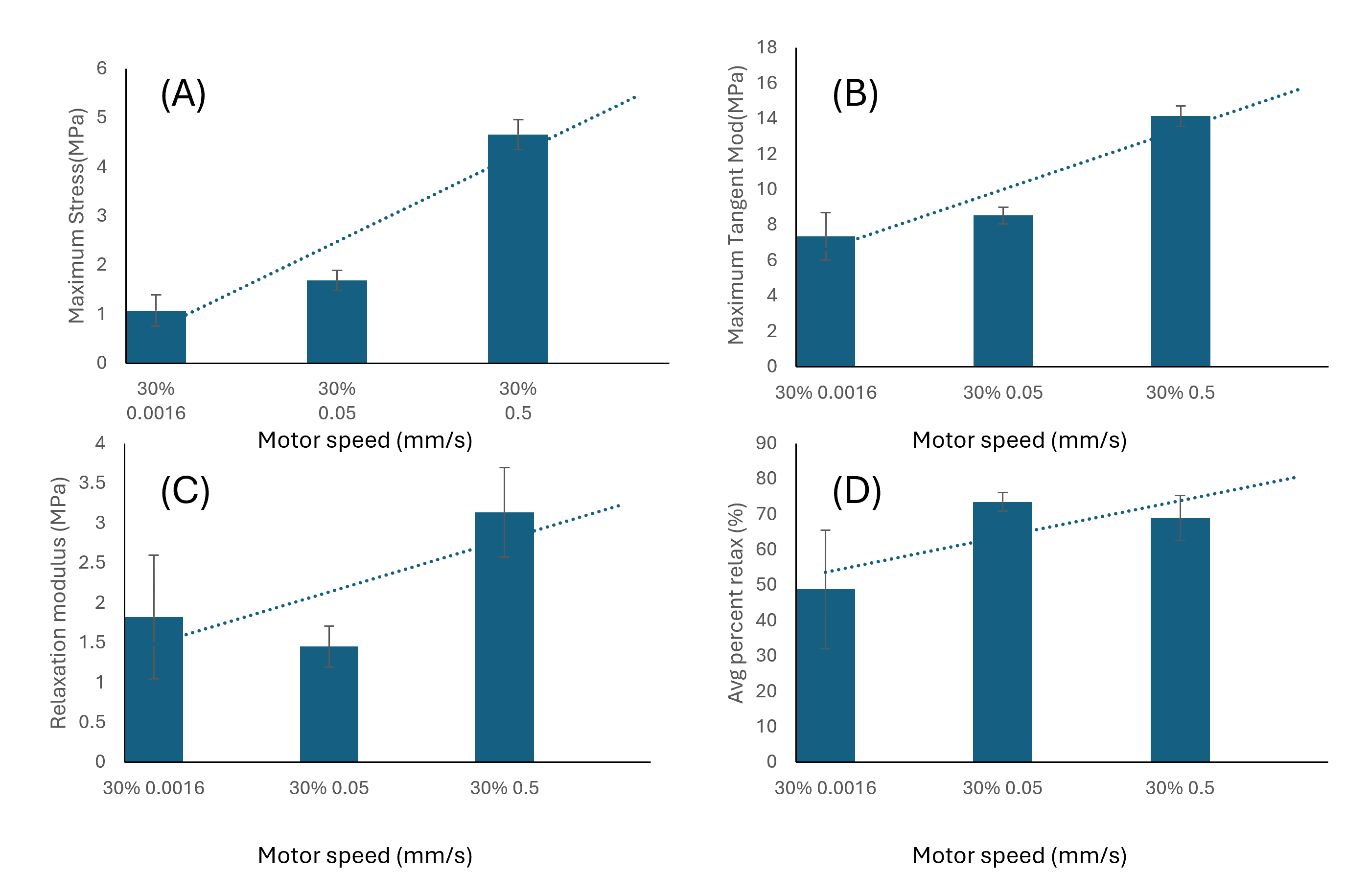


**Figure S11**: *Mechanical parameters from compression tests:* The A) maximum stress, B) maximum tangent modulus, C) relaxation modulus and D) percentage relaxation of the tested samples per strain rate (x-axis: speed of compressing platen). Error bars: standard deviations. Trend-line is linear regression and shows that the highest strain rate (which also led to highest deformation) shows increase modulus and maximum stress, as expected.


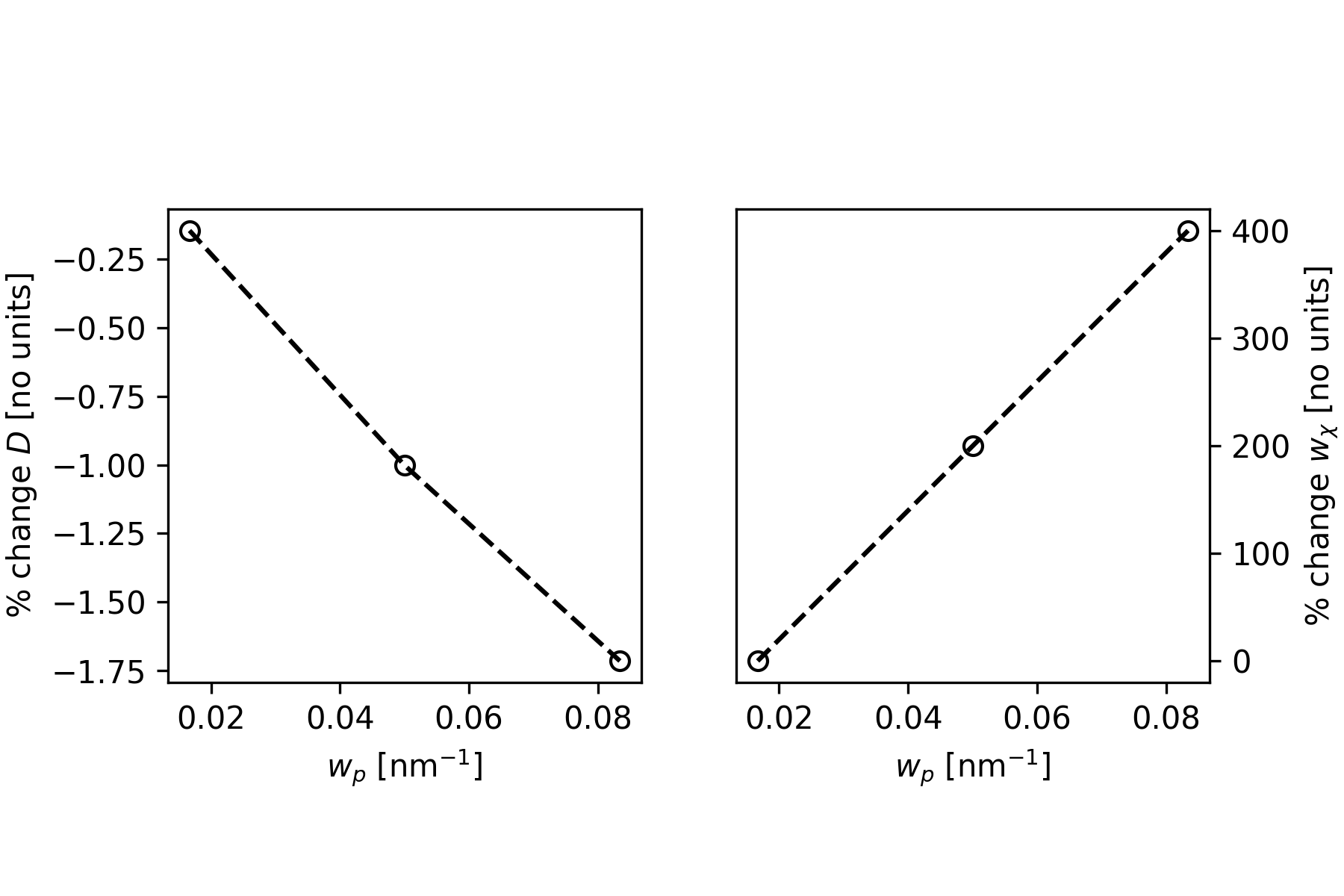


**Figure S12**: *Percentage error in D-period and w_χ_ using Gaussian models*: Supplementing **Figure 2C2** in the main text, the plots above show the percentage errors in estimated (left) D-period and w_χ_ using Gaussian fits, rather than the 3D diffraction model presented here, with the simulated data shown in **Figure 2C2**.

**Supplementary Information References**:

[1] P. Fratzl, in *Lecture Notes of the Introductory Course to ECNS99* (Eds: G. Kadar, L. Rosta), **1999**, pp. 57–72.

[2] Y. Zhang, J. Garrevoet, Y. Wang, J. T. Roeh, N. J. Terrill, G. Falkenberg, Y. Dong, H. S. Gupta, *ACS Nano* **2020**, *14*, DOI 10.1021/acsnano.0c02879.

[3] P. Fratzl, N. Fratzl-Zelman, K. Klaushofer, *Biophys J* **1993**, *64*, 260.

[4] S. R. Inamdar, D. P. Knight, N. J. Terrill, A. Karunaratne, F. Cacho-Nerin, M. M. Knight, H. S. Gupta, *ACS Nano* **2017**, *11*, 9728.

[5] I. Silva Barreto, M. Pierantoni, M. Hammerman, E. Törnquist, S. Le Cann, A. Diaz, J. Engqvist, M. Liebi, P. Eliasson, H. Isaksson, *Matrix Biology* **2023**, *115*, DOI 10.1016/j.matbio.2022.11.006.

[6] H. D. Barth, E. A. Zimmermann, E. Schaible, S. Y. Tang, T. Alliston, R. O. Ritchie, *Biomaterials* **2011**, *32*, DOI 10.1016/j.biomaterials.2011.08.013.

[7] M. Fernández, J. Keyriläinen, R. Serimaa, M. Torkkeli, M. L. Karjalainen-Lindsberg, M. Tenhunen, W. Thomlinson, V. Urban, P. Suortti, *Phys Med Biol* **2002**, *47*, DOI 10.1088/0031-9155/47/4/303.
